# Supplementary material for: Societal cost of nine selected maternal morbidities in the United States
Source: PLoS One. 2022 Oct 26;17(10):e0275656. doi: 10.1371/journal.pone.0275656 (PMC9603953; doi:10.1371/journal.pone.0275656)
Supplement: S1 Appendix — (DOCX) [file pone.0275656.s001.docx]

# S1 Appendix. Description of Literature Review and Search Terms

We developed our model using estimates from Luca et al. that contained the outcomes and associated costs of maternal mental health conditions (MMHCs). We updated the incidence and costs to 2019 data where available.

To expand the model to include more maternal morbidity conditions, we used existing literature on the incidence and prevalence of maternal morbidity conditions, outcomes, and associated medical and nonmedical costs as the primary sources of data. To ensure the model reflected the most relevant and rigorous evidence, we conducted three systematic literature searches: (1) prevalence or incidence of conditions associated with maternal morbidity; (2) likelihood or risk of developing outcomes given the maternal morbidity; and (3) medical and nonmedical associated costs.

When developing the search terms, we sought to capture the costs of maternal morbidity through five years postpartum from both maternal and child perspectives. Accordingly, we excluded certain delivery outcomes, such as abortion and miscarriage, which do not involve postpartum costs.

**Search databases and terms.** We identified original articles published in peer-reviewed scientific journals by searching Ovid MEDLINE, CINAHL, Cochrane Database of Systematic Reviews, American Psychological Association’s (APA) PsycInfo, and EconLit. To obtain current estimates of the prevalence, incidence, and costs of maternal morbidity and related outcomes, we restricted the period of our search to articles published in or after 2010. Table A.1 provides the terms we used by search.

To augment the literature search, we reviewed supplementary studies from four other sources: (1) the references of articles found through our literature search; (2) the references of other reports and publications that identify maternal morbidity outcomes and associated costs; (3) Google Scholar search; and (4) grey literature custom search. For our review of the grey literature, we searched reputable websites such as Centers for Disease Control and Prevention (CDC) and World Health Organization (WHO) for relevant articles. Table A.2 provides the Google Scholar and grey literature search terms we used as well as the specified website list.

**Screening articles.** After conducting the three searches noted above, we reviewed article titles to ascertain relevance to the conceptual model, dropping any irrelevant articles. We then reviewed the abstracts and full text of the remaining articles to determine the articles that contained impact and cost estimates we could use in the model. We included original studies that analyzed maternal morbidity and associated outcomes as the main exposure of interest as well as systematic reviews. We developed the following inclusion criteria to guide our selection of articles during this review:

- **Studies used adequate controls to verify that the reported outcomes occurred because of maternal morbidity exposure rather than potentially confounding factors.** To ensure the high quality of the articles used to inform our model, we included studies that adequately controlled for confounders (such as demographic characteristics or comorbidities) or that used a matched-comparison group design. Included studies had to show at least one significant estimate of the impact of maternal morbidity on outcomes of interest. Finally, studies had to meet high methodological standards to ensure the credibility of estimated impacts.
- **Studies examined outcomes quantifiable in monetary terms.** To quantify the impact of outcomes monetarily for the study to be relevant for our analyses, we excluded outcomes such as reduced frequency of mother–infant interactions and reduced quality of life that cannot be quantified in monetary terms.
- **Studies that examined medical costs were conducted in the United States, with data from 2011 or later.** For example, studies conducted in countries with single-payer or nationalized health care systems, or studies conducted in middle- or low-income countries, might result in different treatment protocols and patterns of health care utilization. In addition, studies that used data from before 2011 might not accurately represent current medical costs given changes in treatment protocols and health care utilization over time.

Across the three searches detailed in Table A.1, we identified 8,337 records through our database search. We also found 95 records through grey literature searches and snowballing. After deduplication, the total number of unique records was 5,365 (Table A.2). Figure 2 (see main article) shows our Preferred Reporting Items for Systematic Reviews and Meta-Analyses (PRISMA) flowchart for this study. It details the number of articles found through each search, those selected for full-text review after screening title and abstracts, and the final number identified for study inclusion. We ultimately identified a total of 224 articles documenting the maternal morbidity conditions, the likelihood of developing any medical outcomes associated with a maternal morbidity condition, the likelihood of a societal consequence, and costs associated with these conditions or outcomes.

**Refining the model.** We initially sought to document the costs of societal consequences resulting from both maternal morbidity conditions and associated outcomes. Upon closer inspection, we decided to model only medical outcomes and societal consequences *directly resulting* from maternal morbidity conditions, which prevented overestimating the effects of maternal morbidity. For example, we did not model productivity loss resulting both from MMHCs (a morbidity in the model) and from preterm birth (a designated outcome) because it would double count any maternal-child pairs that experienced both MMHCs and preterm birth. Thus, many of the articles found during the literature review search were no longer relevant for the model.

We excluded additional articles from our analysis when collecting parameters to use in the model. Reviewing the conceptual model and articles in more detail, we excluded any connections that did not have at least three supporting articles. In addition, we excluded articles detailing conditions that developed before pregnancy, pooled maternal morbidity estimates, and studies that did not contain any usable estimates or any nonsignificant statistical estimates. When there was one or more credible U.S.-based impact estimate for a morbidity-outcome connection, we did not use estimates from other countries. However, if there were no U.S.-based estimates or if the estimate was based on data prior to 2010, we included estimates from the Organisation for Economic Co-operation and Development (OECD) countries as additional support. Figure 2 (see main article) details the number of articles excluded from the model for each reason, and our reference list details the full list of included articles.

We consulted expert advisors throughout our process of reviewing connections to include in the final model and supporting evidence. With their input, we kept certain connections that had fewer than three supporting articles and removed others. This process enabled us to rely on existing evidence to develop our model while following guidance of thought leaders in the field of maternal morbidity.

S1 Table 1. Database search terms used in literature review, by search

| Search | Search terms |
| --- | --- |
| **Search 1 – Prevalence or incidence of maternal morbidity conditions** | |
| Population | mother, delivery, maternity, maternal morbidity, child*, infant, fetus, baby, newborn, neonat*, antenatal, prenatal, postnatal, pregnan* |
| Conditions | acute myocardial infarction, aneurysm, acute renal failure, adult respiratory distress syndrome, amniotic fluid embolism, cardiac arrest/ventricular fibrillation, conversion of cardiac rhythm, disseminated intravascular coagulation, eclampsia, heart failure, arrest during surgery, puerperal cerebrovascular disorders, pulmonary edema, acute heart failure, severe anesthesia complications, sepsis, shock, sickle cell disease with crisis, air and thrombotic embolism, blood products transfusion, hysterectomy, temporary tracheostomy, ventilation, gestational diabetes, obstetric anal sphincter injury, chronic pain, mental health, stress, trauma |
| Prevalence/incidence | prevalence OR incidence |
| **Search 2a – Likelihood of developing outcomes** | |
| Population | mother, delivery, maternity, maternal morbidity, child*, infant, fetus, baby, newborn, neonat*, antenatal, prenatal, postnatal, pregnan* |
| Conditions | acute myocardial infarction, aneurysm, acute renal failure, adult respiratory distress syndrome, amniotic fluid embolism, cardiac arrest/ventricular fibrillation, conversion of cardiac rhythm, disseminated intravascular coagulation, eclampsia, heart failure, arrest during surgery, puerperal cerebrovascular disorders, pulmonary edema, acute heart failure, severe anesthesia complications, sepsis, shock, sickle cell disease with crisis, air and thrombotic embolism, blood products transfusion, hysterectomy, temporary tracheostomy, ventilation, gestational diabetes, obstetric anal sphincter injury, chronic pain, mental health, stress, trauma |
| Outcomes | anemia, asthma, cardiovascular disease, hypertension, heart disease, cystic fibrosis, diabetes, digestive system issues, enteritis, colitis, gastro-osophageal reflux, functional intestinal disorder, hemorrhage, HIV infection, hospitalization rates, readmission rates, infertility, mental illness, behavioral health, depression, anxiety, PTSD, OCD, mortality, multiple sclerosis, pain AND (perineal OR lower abdominal OR pelvic OR lumbar), pulmonary embolism, deep vein thrombosis, uterine prolapse, uterine rupture and scar, urinary incontinence, fecal incontinence, anal incontinence, haemorrhoids, constipation, venous thromboembolism, insomnia, hypersomnia, narcolepsy, fatigue, breastfeed*, developmental issues, intellectual disability, low birth weight, macrosomia, malnutrition, mortality, SIDS, perinatal mortality, preterm birth, stillbirth |
| Impact estimates | odds OR likelihood OR aOR OR risk OR aRR OR hazard ratio OR aHR |
| **Search 2b – Likelihood of societal consequences arising from conditions or outcomes** | |
| Population | - (no restrictions) |
| Conditions | acute myocardial infarction, aneurysm, acute renal failure, adult respiratory distress syndrome, amniotic fluid embolism, cardiac arrest/ventricular fibrillation, conversion of cardiac rhythm, disseminated intravascular coagulation, eclampsia, heart failure, arrest during surgery, puerperal cerebrovascular disorders, pulmonary edema, acute heart failure, severe anesthesia complications, sepsis, shock, sickle cell disease with crisis, air and thrombotic embolism, blood products transfusion, hysterectomy, temporary tracheostomy, ventilation, gestational diabetes, obstetric anal sphincter injury, chronic pain, mental health, stress, trauma |
| Outcomes | anemia, asthma, cardiovascular disease, hypertension, heart disease, cystic fibrosis, diabetes, digestive system issues, enteritis, colitis, gastro-osophageal reflux, functional intestinal disorder, hemorrhage, HIV infection, hospitalization rates, readmission rates, infertility, mental illness, behavioral health, depression, anxiety, PTSD, OCD, mortality, multiple sclerosis, pain AND (perineal OR lower abdominal OR pelvic OR lumbar), pulmonary embolism, deep vein thrombosis, uterine prolapse, uterine rupture and scar, urinary incontinence, fecal incontinence, anal incontinence, haemorrhoids, constipation, venous thromboembolism, insomnia, hypersomnia, narcolepsy, fatigue, breastfeed*, developmental issues, intellectual disability, low birth weight, macrosomia, malnutrition, mortality, SIDS, perinatal mortality, preterm birth, stillbirth |
| Societal consequences | productivity loss*, loss of income, absenteeism, presenteeism, lower labor force participation, reduced productivity, Medicaid, Supplemental Nutrition Assistance Program, SNAP, Special Supplemental Nutrition Program for Women, Infants, and Children, WIC, Temporary Assistance for Needy Families, TANF, criminal, prison, child protective services, foster care, quality of life, special needs, disability, emotional problem, conduct problem, social security disability insurance, SSDI, social care, social service*, low educational attainment |
| Impact estimates | Odds OR likelihood OR aOR OR risk OR aRR OR hazard ratio OR aHR |
| **Search 3a – Costs of outcomes (medical and nonmedical)** | |
| Population | mother, delivery, maternity, maternal morbidity, child*, infant, fetus, baby, newborn, neonat*, antenatal, prenatal, postnatal, pregnan* |
| Outcomes | anemia, asthma, cardiovascular disease, hypertension, heart disease, cystic fibrosis, diabetes, digestive system issues, enteritis, colitis, gastro-osophageal reflux, functional intestinal disorder, hemorrhage, HIV infection, hospitalization rates, readmission rates, infertility, mental illness, behavioral health, depression, anxiety, PTSD, OCD, mortality, multiple sclerosis, pain AND (perineal OR lower abdominal OR pelvic OR lumbar), pulmonary embolism, deep vein thrombosis, uterine prolapse, uterine rupture and scar, urinary incontinence, fecal incontinence, anal incontinence, haemorrhoids, constipation, venous thromboembolism, insomnia, hypersomnia, narcolepsy, fatigue, breastfeed*, developmental issues, intellectual disability, low birth weight, macrosomia, malnutrition, mortality, SIDS, perinatal mortality, preterm birth, stillbirth, low educational attainment |
| Costs | cost*, financ*, economic, payment*, expenditure*, payer, societal burden, societal impact, disease burden |
| **Search 3b – Costs of conditions, outcomes, or societal consequences (medical and nonmedical)** | |
| Population | - (no restrictions) |
| Conditions | acute myocardial infarction, aneurysm, acute renal failure, adult respiratory distress syndrome, amniotic fluid embolism, cardiac arrest/ventricular fibrillation, conversion of cardiac rhythm, disseminated intravascular coagulation, eclampsia, heart failure, arrest during surgery, puerperal cerebrovascular disorders, pulmonary edema, acute heart failure, severe anesthesia complications, sepsis, shock, sickle cell disease with crisis, air and thrombotic embolism, blood products transfusion, hysterectomy, temporary tracheostomy, ventilation, gestational diabetes, obstetric anal sphincter injury, chronic pain, mental health, stress, trauma |
| Outcomes | anemia, asthma, cardiovascular disease, hypertension, heart disease, cystic fibrosis, diabetes, digestive system issues, enteritis, colitis, gastro-osophageal reflux, functional intestinal disorder, hemorrhage, HIV infection, hospitalization rates, readmission rates, infertility, mental illness, behavioral health, depression, anxiety, PTSD, OCD, mortality, multiple sclerosis, pain AND (perineal OR lower abdominal OR pelvic OR lumbar), pulmonary embolism, deep vein thrombosis, uterine prolapse, uterine rupture and scar, urinary incontinence, fecal incontinence, anal incontinence, haemorrhoids, constipation, venous thromboembolism, insomnia, hypersomnia, narcolepsy, fatigue, breastfeed*, developmental issues, intellectual disability, low birth weight, macrosomia, malnutrition, mortality, SIDS, perinatal mortality, preterm birth, stillbirth, low educational attainment |
| Societal consequences | productivity loss*, loss of income, absenteeism, presenteeism, lower labor force participation, reduced productivity, Medicaid, Supplemental Nutrition Assistance Program, SNAP, Special Supplemental Nutrition Program for Women, Infants, and Children, WIC, Temporary Assistance for Needy Families, TANF, criminal, prison, child protective services, foster care, quality of life, special needs, disability, emotional problem, conduct problem, social security disability insurance, SSDI, social care, social service* |
| Cost | cost*, financ*, economic, payment*, expenditure*, payer, societal burden, societal impact, disease burden, cost of illness |

S1 Table 2. Google Scholar and grey literature search terms used in literature review, by search

| Search | Search terms |
| --- | --- |
| **Search A – Grey literature: Overall conceptual model** | |
| Websites | WHO, Gates Foundation, California Health Care Foundation, Association of Maternal and Child Health Programs, Maternal Health Task Force |
| Keywords | maternal morbidity, maternal, pregnant, pregnancy, postpartum, peripartum, child, morbidity, mortality, delivery, illness, infection, chronic or acute disability, disease, neglect, healthcare disparities, nutrition, costs, economic, Medicaid, expenditures, social support, social programs, societal, family impact |
| **Search B.1 – Grey literature: Costs of conditions, outcomes, or societal consequences** | |
| Websites | Commonwealth, IBM (Truven), OPTUM, Kaiser, MACPAC, CDC, MMWR, AHRQ, Milken Institute, Urban Institute, RAND, NORC |
| Keywords (cost) | cost, cost of illness |
| Keywords (conditions, outcomes, and societal consequences) | amniotic fluid embolism, dyspareunia, hemorrhage, hyperemesis gravidarum, maternal morbidity, obstetric anal sphincter injury, pelvic floor disorder, pre-eclampsia, eclampsia, renal disease, sepsis, thyroid disorders, traumatic birth experience, acute office-based visit, amniotic fluid embolism, antepartum hemorrhage, conduct disorder, dental visit, emergency department visit, episiotomy, fetal distress, fetal malformations, hemorrhage, hyperbilirubinemia, hypertensive disorders, hypoglycemia, infant mortality, child mortality, infection, inpatient visit, instrumental delivery, intellectual disability, intensive care unit, jaundice, kidney dysfunction, large for gestational age, macrosomia, maternal mortality, neurodevelopmental disorder, neuropsychiatric morbidity, neonatal intensive care unit, obstructive sleep apnea, outpatient visit, overactive bladder, pelvic organ prolapse, perineal trauma, poor fetal growth, preterm labor, preventative visit, respiratory distress, small for gestational age, venous thromboembolism, homelessness, lower educational attainment, school absences, educational disruption, prison, criminal justice, child protective services, educational needs, productivity loss, absenteeism, social services |
| **Search B.2 – Google Scholar: Costs of conditions, outcomes, or societal consequences** | |
| Websites | Google Scholar |
| Keywords (cost) | cost, cost of illness |
| Keywords (conditions, outcomes, and societal consequences) | amniotic fluid embolism, dyspareunia, hemorrhage, hyperemesis gravidarum, maternal morbidity, obstetric anal sphincter injury, pelvic floor disorder, pre-eclampsia, eclampsia, renal disease, sepsis, thyroid disorders, traumatic birth experience, acute office-based visit, amniotic fluid embolism, antepartum hemorrhage, conduct disorder, dental visit, emergency department visit, episiotomy, fetal distress, fetal malformations, hemorrhage, hyperbilirubinemia, hypertensive disorders, hypoglycemia, infant mortality, child mortality, infection, inpatient visit, instrumental delivery, intellectual disability, intensive care unit, jaundice, kidney dysfunction, large for gestational age, macrosomia, maternal mortality, neurodevelopmental disorder, neuropsychiatric morbidity, neonatal intensive care unit, obstructive sleep apnea, outpatient visit, overactive bladder, pelvic organ prolapse, perineal trauma, poor fetal growth, preterm labor, preventative visit, respiratory distress, small for gestational age, venous thromboembolism, homelessness, lower educational attainment, school absences, educational disruption, prison, criminal justice, child protective services, educational needs, productivity loss, absenteeism, social services |
| **Search C.2 – Google Scholar: Likelihood of societal consequences arising from conditions or outcomes** | |
| Websites | Google Scholar |
| Keywords (societal consequences) | productivity, income, absenteeism, labor force, SNAP, WIC, TANF, welfare, social services, criminal, SSDI, educational attainment |
| Keywords (conditions and outcomes) | amniotic fluid embolism, blood clotting disorders, cardiovascular, dyspareunia infection, sepsis, maternal morbidity, pelvic floor disorder, eclampsia, severe maternal morbidity, traumatic birth experience, office-based visit, anal incontinence, caesarean section, cesarean section, cancer child obesity, child overweight, dental visit, emergency department visit, fetal distress, fetal malformation, hospitalization, hyperbilirubinemia, instrumental delivery, jaundice, kidney dysfunction, macrosomia, mortality, neonatal intensive care unit, outpatient visit, overactive bladder, pelvic organ prolapse, perineal trauma, poor fetal growth, preterm birth, preterm labor, preventative visits, respiratory distress, visual impairment, hyperemesis gravidarum, pre-eclampsia, thyroid, cerebral palsy, episiotomy, hypoglycemia, inpatient visits, neurodevelopmental, neuropsychiatric morbidity, stillbirth |
