# Supplementary material for: Societal cost of nine selected maternal morbidities in the United States
Source: PLoS One. 2022 Oct 26;17(10):e0275656. doi: 10.1371/journal.pone.0275656 (PMC9603953; doi:10.1371/journal.pone.0275656)
Supplement: S2 Appendix — (DOCX) [file pone.0275656.s002.docx]

# S2 Appendix. Prevalence of Maternal Morbidity Conditions and Outcomes

S2 Table 1. Prevalence of maternal morbidity conditions and outcomes

| **Measure** | **Estimate** | **Population** | **Data Year** | **Data Source** | **Citation** |
| --- | --- | --- | --- | --- | --- |
| Maternal Morbidity Conditions | | | | | |
| Amniotic fluid embolism/ complications | 5.5 per 100,000 (5.5–5.5) | Population databases from five countries. | Years vary by country, ranging from 1991–2010 | Population databases from five countries | (Knight et al., 2012) |
| Cardiac arrest/ventricular fibrillation | 8.5 per 100,000 (7.7–9.3) | U.S. hospitalizations for delivery | 1998–2011 | Nationwide Inpatient Sample | (Mhyre et al., 2014) |
| Gestational diabetes mellitus | 6.9% (6.88%–6.93%) | U.S. births | 2019 | National Vital Statistics System | (Martin et al., 2021) |
| Hemorrhage | 10.5%–34.4% | U.S. hospitalizations for delivery | 2009 | Nationwide Inpatient Sample | (Stranges et al., 2012) |
| Hypertensive disorders (including preeclampsia and eclampsia) | 6.61% (6.58%–6.64%) | Live births in the U.S. | 2017 | U.S. birth certificate data | (Butwick et al., 2020) |
| MMHCs | 13.2% (12.6%–13.8%) | Women with a live birth | 2018 | Pregnancy Risk Assessment Monitoring System (PRAMS) | (Bauman et al., 2020) |
| Renal disease | 0.4% (0.035%–0.042%) | Women between 15–49 with a live–birth delivery | 2007–2011 | Truven Health MarketScan Commercial Claims and encounters database | (Law et al., 2015) |
| Sepsis | 9.8 per 10,000 (9.4–10.3) | Live births in California | 2005–2007 | Vital statistics records | (Acosta et al., 2013) |
| Venous thromboembolism | 1.99 per 1,000 (1.52-2.46) | Pregnancy–related hospitalizations | 2006–2009 | Nationwide Inpatient Sample | (Ghaji et al., 2013) |
| Maternal Outcomes | | | | | |
| Prevalence of SNAP receipt | 46.7% | U.S. households with children under 18 | 2019 | American Community Survey | (U.S. Census Bureau, 2021a) |
| Prevalence of WIC receipt | 26.3% | Pregnant and postpartum women ages 15–44 | 2017 | Current Population Survey | (U.S. Department of Agriculture, 2020) |
| Prevalence of TANF receipt | 2.53% | U.S. families with children under 18 years | FY2019 | TANF program data; American Community Survey | (U.S. Department of Health and Human Services, 2020) |
| Prevalence of Medicaid receipt | 42.1% | Births to women in 2019 | 2019 | National Vital Statistics System | (Martin et al., 2020) |
| Incidence of cesarean section | 31.7% | Births to women in 2019 | 2019 | National Vital Statistics System | (Martin et al., 2021) |
| Maternal mortality rate | 20.1 per 100,000 | Births to women in 2019 | 2019 | National Vital Statistics System | (Hoyert, 2021) |
| Average length of peripartum stay | 2.6 days | U.S. delivering women | NA | CMS | (CMS, n.d.) |
| Labor force participation rate | 63.8% | Women, 16 years and older, with children under 3 years old | 2019 | Current Population Survey | (U.S. Bureau of Labor Statistics, 2021) |
| Unemployment rate | 4.0% | Women, 16 years and older, with children under 3 years old | 2019 | Current Population Survey | (U.S. Bureau of Labor Statistics, 2021) |
| Incidence of pregnancy-associated stroke | 0.012% | U.S. women, ages 15 to 54 years, admitted for delivery from 1998 to 2014 | 1998–2014 | Nationwide Inpatient Sample | (Miller et al., 2020) |
| Incidence of suicide | 6.4 per 100,000 women | U.S. women | 2018 | CDC Multiple Cause of Death Files | (United Health Foundation, 2020) |
| Child Outcomes | | | | | |
| Prevalence of asthma | 2.6% | U.S. children ages 0–4 | 2019 | National Health Interview Survey | (CDC, 2020d) |
| Rate of exclusive breastfeeding through 3 months | 46.9% | Households with children aged 19–35 months | Children born in 2017 | National Immunization Survey | (CDC, 2020c) |
| Prevalence of critical congenital heart defects | 19.93 per 10,000 live births | 39 U.S. population-based birth defects surveillance programs | 2010–2014 | National Birth Defects Prevention Network (NBDPN) annual data report | (Mai et al., 2019) |
| Incidence of child mental disorders | 17.4% | Noninstitutionalized children aged 0–16 years in the U.S. | 2016 | National Survey of Children’s Health | (Cree et al., 2018) |
| Incidence of type 1 diabetes mellitus | 22.3 per 100,000 | Youths < 20 years | 2014–2015 | SEARCH for Diabetes in Youth Study | (Divers et al., 2020) |
| Rate of ED visits | 58.4 visits per 100 children | U.S. children ages 1–4 | 2018 | National Hospital Ambulatory Medical Care Survey | (Cairns et al., 2018) |
| Incidence of fetal abnormalities | 26.2% | Newborns born to women ages 15–49 | 2007–2011 | Truven Health MarketScan Commercial Claims and Encounters database | (Law et al., 2015) |
| Incidence of neonatal hypoglycemia | 10.0% | Referenced studies use samples from the University of Iowa Hospitals and Clinics (Heck, 1987) and a rural tertiary care center in Bangalore (Samayam, 2015) | Authors reference two studies for this estimate; one from 1987 and another from 2015. | – | (Thompson-Branch & Havranek, 2017, p.) |
| Incidence of neonatal sepsis | 516–970 per 100,000 live births | Neonates and children | 1995–2005 | Multiple observational studies on the incidence of sepsis in children | (Fleischmann-Struzek et al., 2018) |
| Rate of nonfatal injuries resulting in ED visit | 7.89% | U.S. children ages 0– 4 | 2019 | WISQARS Cost of Injury Reports on Nonfatal, unintentional injuries | (CDC, 2020b) |
| Prevalence of obesity | 13.4% | U.S. children ages 2–5 | 2017–2018 | National Health and Nutrition Examination Surveys | (Fryar et al., 2020) |
| Incidence of poor fetal growth | 25.9 per 1,000 deliveries | Delivering women in the U.S. | 2014 | National Inpatient Sample | (Fingar et al., 2017) |
| Preterm birth | 10.2% | U.S. births | 2019 | National Vital Statistics System | (Martin et al., 2021) |
| Incidence of pediatric acute respiratory distress syndrome | 3.26% | 145 pediatric intensive care units (PICUs) from 27 countries | 2016–2017 | Multiple international study sites | (Khemani et al., 2019) |
| SIDS death rate | 35.13 deaths per 100,000 live births | Births to women in 2018 | 2018 | NCHS CDC WONDER database | (CDC, 2020a) |
| Fetal death | 587.8 fetal deaths per 100,000 live births and fetal deaths | Live births and fetal deaths | 2015–2017 | National Vital Statistics System | (Hoyert & Gregory, 2020) |
| Likelihood of attending a well-child visit | 65% | Children ages 0–6 | 2011–2016 | Research consortium composed of the OCHIN network and the Virginia Commonwealth University Health System (VCUHS) | (Wolf et al., 2018) |

Notes: ED = emergency department; MMHCs = maternal mental health conditions; SIDS = sudden infant death syndrome; SNAP = Supplemental Nutrition Assistance Program; TANF = Temporary Assistance for Needy Families; WIC = Special Supplemental Nutrition Program for Women, Infants, and Children.
