# Supplementary material for: Societal cost of nine selected maternal morbidities in the United States
Source: PLoS One. 2022 Oct 26;17(10):e0275656. doi: 10.1371/journal.pone.0275656 (PMC9603953; doi:10.1371/journal.pone.0275656)
Supplement: S3 Appendix — (DOCX) [file pone.0275656.s003.docx]

# S3 Appendix. Studies and Data Sources Used to Inform the Cost Estimates Used in the Model

S3 Table 1. Studies and data sources used to inform cost estimates used in the model

| **Parameter** | **Sample Years** | **Estimate (in 2019 $)** | **Cost Type** | **Methodology** | **Citation/Data Source** |
| --- | --- | --- | --- | --- | --- |
| Maternal Outcomes | | | | | |
| Annual cost per case of SNAP | 2019 | $1,691 | Nonmedical | We calculated the cost per person on SNAP from the total issuance and administrative costs of SNAP in the U.S., divided by the total number of participants. | (U.S. Food and Drug Administration, 2017) |
| Annual cost per case of WIC | 2019 | $816 | Nonmedical | We calculated the cost per person on WIC from the total issuance and administrative costs of WIC, divided by the total number of participants. | (U.S. Department of Agriculture, 2019c) |
| Annual cost per case of Medicaid | 2014 | $7,883 | Nonmedical | We used the CMS estimates for the cost per Medicaid per enrollee for the total U.S., found in Table 26. | (Centers for Medicare and Medicaid Services, 2017) |
| Annual cost per case of TANF | 2016 | $10,374 | Nonmedical | We calculated the cost per TANF case by dividing total federal TANF and state maintenance-of-effort expenditures, including both services and administrative costs, by the average number of TANF recipients. | (U.S. Department of Health and Human Services, 2018; U.S. Department of Health and Human Services, 2017) |
| Incremental cost of cesarean section | 2010 | $12,179 | Medical | U.S. estimates are pulled from Tables 56 and 57 (Nationally Weighted Average Charges and Payments Combining All Phases of Care and for Each Individual Phase of Care by Type of Service for Vaginal and Cesarean Childbirths, 2010 Commercial and 2010 Medicaid) Truven Health Analytics MarketScan study for Childbirth Connection, Catalyst for Payment Reform, and the Center for Healthcare Quality and Payment Reform (2013). | (Corry et al., 2013) |
| Medical cost of maternal mortality | 2013 | $3,406 | Medical | We obtained the direct costs of suicide, including ambulance transport, a coroner or medical examination, an emergency department visit, inpatient hospitalization, and nursing home care from Shepard et al (2017). | (Shepard et al., 2016) |
| Annual nonmedical cost of maternal mortality | 2019 | $42,900 | Nonmedical | We calculated the indirect costs of maternal mortality by estimating the annual salary of women with children under age 6, based on data from BLS. | (U.S. Bureau of Labor Statistics, 2020) |
| Cost of per day for inpatient stay | 2018 | $2,723 | Medical | We used the daily cost of an inpatient stay, for women, as a proxy for the cost of an additional day of a peripartum stay. | (AHRQ, n.d.) |
| Cost per unemployed mother | 2019 | $42,900 | Nonmedical | U.S. estimates of median usual weekly earnings of women of any marital status with children under 6 years are pulled from Table 7. State earnings estimates are pulled from Table 1. We assumed that women work and/or are paid for working 52 weeks per year, and multiplied the median weekly earnings found in the BLS report by 52 weeks. | (U.S. Bureau of Labor Statistics, 2020) |
| Cost of per day for inpatient stays with a diagnosis related to stroke | 2018 | $20,719 | Medical | This cost estimate is the mean hospital inpatient cost for female patients in 2018 that were hospitalized with a diagnosis (defined by the Medicare-Severity Diagnosis Related Groups, MS-DRG) of #061 Acute ischemic stroke with use of thrombolytic agent with major complication or comorbidity, #062 Acute ischemic stroke w use of thrombolytic agent with complication or comorbidity, or #063 Acute ischemic stroke w use of thrombolytic agent without complication or comorbidity. | (AHRQ, n.d.) |
| Medical cost of suicide | 2013 | $3,406 | Medical | We obtained the direct costs of suicide, including ambulance transport, a coroner or medical examination, an emergency department visit, inpatient hospitalization, and nursing home care from Shepard et al (2017). | (Shepard et al., 2016) |
| Annual nonmedical cost of suicide | 2019 | $42,900 | Nonmedical | We calculated the indirect costs of maternal mortality by estimating the annual salary of women with children under age 6, based on data from BLS. | (U.S. Bureau of Labor Statistics, 2020) |
| Child Outcomes | | | | | |
| Annual medical cost per case of asthma | 2007–2013 | $955 | Medical | We based the annual medical cost per case of asthma on the results from Sullivan et al. (2017). | (Sullivan et al., 2017) |
| Annual nonmedical cost per case of asthma | 2008–2012 | $425 | Nonmedical | We based the annual nonmedical cost per case of asthma on the cost of absenteeism per child from Nurmagambetov et al., 2017. | (Nurmagambetov et al., 2017) |
| Annual cost of suboptimal breastfeeding | 2007 | $2,652 | Medical | We based the incremental cost due to suboptimal breastfeeding on Bartick and Reinhold (2010), who estimated the excess cost of suboptimal breastfeeding, relative to the costs if 80% of U.S. families could comply with the recommendation to exclusively breastfeed for 6 months. Excess costs included necrotizing enterocolitis, otitis media, gastroenteritis, hospitalization for lower respiratory tract infections, atopic dermatitis, SIDs, childhood asthma, childhood leukemia, type 1 diabetes mellitus, and childhood obesity. To avoid double-counting, we excluded the costs due to SIDS, asthma, and childhood obesity. | (Bartick and Reinhold, 2010) |
| Annual costs of cardio conditions | 2012 | $58,842 | Medical | We calculated this cost by taking the total inpatient costs for children with congenital heart disease in 2012 ($6,722 million), divided by the total number of children with congenital heart disease and a hospital discharge in 2012 (138,797). | (Faraoni et al., 2016) |
| Incremental annual cost per child with behavioral and developmental disorders | 2005–2006 | $12,990 | Nonmedical | We based our estimate on Beecham et al. (2014) who examined the costs of child mental illness among young children. Incremental costs include health and mental health care, education, social care, parents’ out-of-pocket expenses, parents’ absence from work, and accommodation (excluding parental home). | (Kancherla et al., 2012) |
| Annual medical cost of diabetes | 2009-2012 | $15,649 | Medical | We based our estimates on Lee et al. (2015); we summed the median annualized expenditures for hospitalizations, ED, outpatient, insulin, testing, pump for ages 0–4. | (Lee et al., 2015) |
| Annual medical costs emergency room services | 2015 | $805 | Medical | Estimate is from the 2015 Full Year Person-Level File for children aged 0–5, which includes both the ER facility fee and separately-billed doctor expenditures. To obtain a reasonable estimate of mean ED expenditures and remove noise created by surveyed patients who did not visit the ED, we restricted the data to expenditures of at least $100. | (AHRQ, 2019) |
| Annual medical costs for fetal malformations | 2008–2012 | $64,153 | Medical | We added up the admission costs for all congenital anomalies, except for cardiovascular disease (to avoid double counting) and divided this by the number of infants admitted with congenital anomalies (again, except with cardiovascular diseases). | (Faraoni et al., 2016) |
| Medical costs for hypoglycemia | 2015 | $20,500 | Medical | This cost estimate is the mean hospital inpatient cost for infants that were hospitalized with a diagnosis (defined by the ICD–9) of 775.6 neonatal hypoglycemia. | (AHRQ, n.d.) |
| Medical costs for infections | 2018 | $10,059 | Medical | This cost estimate is the weighted average of mean hospital inpatient cost for patients age <1 and ages 1–17 in 2018 that were hospitalized with a diagnosis (Clinical Classification Software Refined, CCSR) of INF002 Septicemia. | (AHRQ, n.d.) |
| Cost per case of nonfatal injuries in children resulting in a hospitalization | 2010 | $8,018 | Medical | We averaged the lifetime cost of a nonfatal injury that resulted in a hospitalization to children ages 0 through 5 who were hospitalized by an average life expectancy of 78.2 years to estimate the annual cost per child injury. Hospitalization costs are considered separately in the CDC database we used (Web-based Injury Statistics Query and Reporting System [WISQARS]). | (CDC, 2019) |
| Annual medical cost of obesity | 2012 | $200 | Medical | Most conservative estimate: Incremental lifetime medical cost of a 10-year-old obese child relative to a 10-year-old normal-weight child who may eventually gain weight ($12,660), assuming an average life expectancy of 78.7 years. Does not consider variation in medical costs throughout the lifetime. | (Finkelstein et al., 2014) |
| Medical costs for poor fetal growth | 2015 | $8,562 | Medical | This cost estimate is the mean hospital inpatient cost for infants that were hospitalized with a diagnosis (defined by the ICD–9) of 764.08 Light For Dates (2000–2499G). | (AHRQ, n.d.) |
| Annual medical cost of preterm birth | 2012 | $78,052 | Medical | We based this estimate off Hall and Greenberg (2016). We divided their total estimated excess costs attributable to preterm birth by the number of preterm birth cases. | (Hall & Greenberg, 2016) |
| Medical costs for respiratory distress syndrome | 2015 | $72,335 | Medical | This cost estimate is the mean hospital inpatient cost for infants that were hospitalized with a diagnosis (defined by the ICD-9) of 769 Respiratory distress syndrome in newborn. | (AHRQ, n.d.) |
| Cost per case of SIDS | 2014 | $23,691 | Nonmedical | Estimates were pulled from Fox et al. (2014). Costs include funeral expenses, outside help (over 6 months), out-of-pocket prescription costs, leave days (over 6 months), absenteeism, ability to perform job at 30 days vs. 6 months, and presenteeism. | (Fox et al., 2014) |
| Medical cost per case of stillbirth | 2013 | $9,611 | Medical | We based our estimate off Heazell et al. (2016). For direct medical costs, we used the study’s estimate that was based on research done with U.S. data and published after 2011. | (Heazell et al., 2016) |
| Nonmedical cost per case of stillbirth | 2013 | $3,448 | Nonmedical | We based our estimate off Heazell et al. (2016). For nonmedical costs, we used the lower bound estimates for funerals, burials plots, and memorials related to stillborn births. | (Heazell et al., 2016) |
| Cost per well-child care visit | 2015 | $181 | Medical | We used an estimate of the median expenses for an office-based provider visit for a child ages 0–5 with a perceived health status of Excellent, Very Good, Good, or Fair. | (AHRQ, 2015) |

Notes: ED = emergency department; SIDS = sudden infant death syndrome; SNAP = Supplemental Nutrition Assistance Program; TANF = Temporary Assistance for Needy Families; WIC = Special Supplemental Nutrition Program for Women, Infants, and Children.
