# Supplementary material for: Societal cost of nine selected maternal morbidities in the United States
Source: PLoS One. 2022 Oct 26;17(10):e0275656. doi: 10.1371/journal.pone.0275656 (PMC9603953; doi:10.1371/journal.pone.0275656)
Supplement: S4 Appendix — (DOCX) [file pone.0275656.s004.docx]

# S4 Appendix. Evidence of Association between Maternal Morbidity Conditions and Maternal and Child Outcomes

S4 Table 1. Evidence of Association between Maternal Morbidity Conditions and Maternal and Child Outcomes

| **Condition** | **Outcome** | **Estimate (95% CI)** | **Study Population** | **Sample Years** | **Sample Size** | **Study Methods** | **Citation** |  |
| --- | --- | --- | --- | --- | --- | --- | --- | --- |
| Maternal Outcomes | | | | | | | |  |
| Gestational diabetes mellitus | Cesarean section | RR = 1.37 (1.35–1.38) | Women ages 18–45 with ascertainable diabetes status and continuous health plan enrollment at least 21 months before and 3 months after the birth | 2004–2011 | n = 839,792 | Retrospective claims analysis examining relative risk of costs and complications in pregnant patients with and without diabetes. | (Jovanovič et al., 2015) |  |
| MMHCs | Cesarean section | OR = 1.34 (1.30–1.37) | Nationwide Inpatient Sample | 1998–2005 | n = 3,215,6438 | Multivariable regression analysis examining delivery-related hospitalizations for select maternal and fetal outcomes by depression diagnosis. | (Bansil et al., 2010) |  |
| MMHCs | Cesarean section | Adjusted OR = 1.59 (1.04–2.42) | Mother–newborn pairs with deliveries in the Beaumont Health System | 2013–2014 | n = 15,492 | Multinomial logistic regression compared each psychiatric diagnosis group to the unaffected referent pregnancies to calculate adjusted odds ratios controlling for baseline differences. | (Ogunyemi et al., 2018) |  |
| MMHCs | Cesarean section | OR = 1.46 (1.02–2.09) | Mother-newborn pairs with deliveries at the Penn State Milton S. Hershey Medical Center in Hershey, PA | 2006–2009 | n = 1,154 | Associations of positive anxiety and depression screens at baseline with each other, demographic, maternity nursery stay-related variables, and health care use were assessed by using Chi-squared tests. A multivariable regression model was built to determine independent association with a positive anxiety screen at baseline. The relationship between anxiety and depression screen findings at baseline with breastfeeding duration was analyzed by using Kaplan-Meier methods. | (Paul et al., 2013) |  |
| Amniotic fluid embolism | Maternal mortality | Case fatality rate = 11%–43% | Population databases from five countries. | Years vary by country, ranging from 1991–2010. | Data from five countries | Review of population databases and validated case ation in five countries (Australia, Canada, the Netherlands, the United Kingdom, the U.S.). | (Knight et al., 2012) |  |
| Cardiac arrest | Maternal mortality | Case fatality rate = 42% (30–55%) | All women who received basic life support in pregnancy in the UK. | 2011–2014 | n = 66 | Prospective, descriptive study using data from the UK Obstetric Surveillance System. Authors used logistic regression to examine associations between predictors and survival after cardiac arrest. | (Beckett et al., 2017) |  |
| Hypertensive disorders | Maternal mortality | Case fatality rate = 2.16 per 10,000 (gestational hypertension) – 5.12 per 10,000 (pre-eclampsia) | All live birth and stillbirth hospital deliveries among women in Ontario, Canada. | 2002–2017 | n = 1,953,943 | Population-based cohort study that calculated relative risks of maternal mortality using modified Poisson regression with a robust error variance. | (Ray et al., 2018) |  |
| Renal disease | Maternal mortality | Case fatality rate = 0.278% (0.138%–0.418%) | All live birth and stillbirth hospital deliveries among women in Ontario, Canada. | 2002–2017 | n = 1,953,943 | Population-based cohort study that calculated relative risks of maternal mortality using modified Poisson regression with a robust error variance. | (Ray et al., 2018) |  |
| Sepsis | Maternal mortality | Fatality rate from sepsis among all pregnant women = 1.13 (0.77–1.67) per 100,000 | Women in the UK | 2006–2008 | n = 2,291,484 | Authors cited the maternal sepsis fatality rate from a 2011 publication in BJOG, which explores causes of maternal death using UK national databases. We used the confidence interval cited in the publication to calculate the sample size. | (Cordioli et al., 2013) |  |
| Venous thrombo-embolism | Maternal mortality | Case fatality rate = 0.32%–1.91% | Multiple (review) | Studies published between 2000 and 2012 | 20 studies in the final review | Systematic review of cohort and case-control studies exploring the epidemiology and economic burden of pregnancy-related VTE. We used the range of estimates for the VTE case fatality rate, omitting the lowest estimate of 0.0000. | (Kourlaba et al., 2016) |  |
| MMHCs | Peripartum stay | An elevated CES-D was associated with a longer peripartum stay of 0.26 (0.04–0.48) days. | Pregnant women recruited from obstetrics clinics in Michigan | 1999–2003 | n = 867 | Multivariable Poisson regression models was used to evaluate predictors of length of stay, adjusting for sociodemographic, antepartum, and obstetric factors. | (Lancaster et al., 2010) |  |
| MMHCs | Unemploy-ment | Individuals with depression have a 20–40% greater likelihood of unemployment. We used the mean (30%) for the main model and the range for the sensitivity analyses. | Multiple (review) | Studies published from 2002 to 2007 | Number of studies not specified | A review of population-based, workplace, and clinical articles reporting on the magnitude and/or nature of depression’s impact on work. | (Lerner and Henke, 2008) |  |
| MMHCs | Absentee-ism | Average annual cost of absenteeism per mother with depression is $619 (adjusted to 2019 $). | Low-income mothers ages 18– 35 and being unmarried, receiving Medicaid, or having incomes less than 300% of the federal poverty level | 2006– 2011 | n = 20,531 | Analysis of the likelihood of employment and workdays missed due to major depressive disorder among mothers using data from the Medical Expenditure Panel Survey, using logistic models controlling for comorbidities, demographics, region, and year. Although their focus was on low-income mothers, the authors also projected cost estimates to the aggregate population, which are the estimates we used in our model. | (Ammerman et al., 2016) |  |
| MMHCs | Absentee-ism | Annual cost of absenteeism per worker with depression is $410 (adjusted to 2019 $). | Workers in 8 countries, including the U.S. | 2012 | n = 1,000 per country | Secondary analysis on data collected in the Global IDEA (Impact of Depression in the Workplace in Europe Audit) survey to examine the effects of depression on presenteeism and absenteeism across 8 countries, controlling for country-specific contextual factors and other factors associated with the outcomes. We used the U.S.-specific estimates to inform the model. | (Evans-Lacko & Knapp, 2016) |  |
| MMHCs | Absentee-ism | Average annual cost of absenteeism per mother with depression is $899 (adjusted to 2019 $). | Depressed patients | 1996– 1997 | n = 479 | Randomized trial examining the impact of improved primary care depression management on absenteeism and presenteeism. A total of 479 patients were recruited from 12 community primary care practices across the U.S., and depression was measured as reporting 5 or more of the 9 Diagnostic and Statistical Manual–III revised criteria for major depression in the past 2 weeks. Absenteeism was measured as the total number of work hours lost due to illness or doctor visits over the past 4 weeks. | (Rost et al., 2004) |  |
| MMHCs | Absentee-ism | Average annual cost of absenteeism per person with depression is $1,792 (adjusted to 2019 $). | Individuals aged 16–64 | 2005 and 2010 | n = 1,461,640 | Case-control study where individuals were matched 1-1 with controls using propensity score matching methods and national survey and administrative claims data from 2005 and 2010 to estimate the incremental burden of individuals with major depressive disorder. We used the 2010 estimates to inform the model. | (Greenberg et al., 2015) |  |
| MMHCs | Presentee-ism | Annual cost of presenteeism per worker with depression is $6,337 (adjusted to 2019 $). | Workers in 8 countries, including the U.S. | 2012 | n = 1,000 per country | Secondary analysis on data collected in the Global IDEA (Impact of Depression in the Workplace in Europe Audit) survey to examine the effects of depression on presenteeism and absenteeism across 8 countries, controlling for country-specific contextual factors and other factors associated with the outcomes. We used the U.S.-specific estimates to inform the model. | (Evans-Lacko & Knapp, 2016) |  |
| MMHCs | Presentee-ism | Average annual cost of presenteeism per mother with depression is $2,879 (adjusted to 2019 $). | Depressed patients | 1996– 1997 | n = 479 | Randomized trial examining the impact of improved primary care depression management on absenteeism and presenteeism. A total of 479 patients were recruited from 12 community primary care practices across the U.S., and depression was measured as reporting 5 or more of the 9 Diagnostic and Statistical Manual–III revised criteria for major depression in the past 2 weeks. Absenteeism was measured as the total number of work hours lost due to illness or doctor visits over the past 4 weeks. | (Rost et al., 2004) |  |
| MMHCs | Presentee-ism | Average annual cost of presenteeism per person with depression is $5,804 (adjusted to 2019 $). | Individuals ages 16–64 | 2005 and 2010 | n = 1,461,640 | Case-control study where individuals were matched 1-1 with controls using propensity score matching methods and national survey and administrative claims data from 2005 and 2010 to estimate the incremental burden of individuals with major depressive disorder. We used the 2010 estimates to inform the model. | (Greenberg et al., 2015) |  |
| Hypertensive disorders | Stroke | RR = 1.16 - 2.80 | Multiple (review) | Studies published between 2005 and 2015. | 22 studies in the final review | Random effects meta-analysis exploring the risk of cardiovascular diseases due to preeclampsia. We omitted the highest estimate, which was an outlier with a large confidence interval from a non-OECD country. | (Wu et al., 2017) |  |
| MMHCs | Suicide | The standardized mortality ratio for suicide for females with unipolar disorder was estimated to be 27.0. (20.9 for males) | Sweden psychiatric inpatient sample | 1973–1995 | n = 15,829 males and 23,353 females | Standardized mortality ratios by 5-year interval age at admission and time of follow-up were calculated using Poisson regression methods, controlling for calendar time of the first admission. | (Ösby et al., 2001) |  |
| MMHCs | SNAP receipt | Probit coefficient = 0.23 (0.14–0.32) | Early Childhood Longitudinal Survey | 2001–2003 | n = 7,900 | Analysis of effects of maternal depression (assessed when the child was 9 months old) on benefit receipt (measured when the child is 2), using multivariate probit models, controlling for age, race/ethnicity, educational attainment, prenatal health, and family history of depression. | (Noonan et al., 2016) |  |
| MMHCs | WIC receipt | Probit coefficient = 0.18 (0.09–0.26) | Early Childhood Longitudinal Survey | 2001–2003 | n = 7,900 | Analysis of effects of maternal depression (assessed when the child was 9 months old) on benefit receipt (measured when the child is 2 years of age), using multivariate probit models, controlling for age, race/ethnicity, educational attainment, prenatal health, and family history of depression. | (Noonan et al., 2016) |  |
| MMHCs | Medicaid receipt | Probit coefficient = 0.24 (0.15–0.34) | Early Childhood Longitudinal Survey | 2001–2003 | n = 7,900 | Analysis of effects of maternal depression (assessed when the child was 9 months old) on benefit receipt (measured when the child is 2) years of age, using multivariate probit models, controlling for age, race/ethnicity, educational attainment, prenatal health, and family history of depression. | (Noonan et al., 2016) |  |
| MMHCs | TANF receipt | Probit coefficient = 0.20 (0.09–0.31) | Early Childhood Longitudinal Survey | 2001–2003 | n = 7,900 | Analysis of effects of maternal depression (assessed when the child was 9 months old) on benefit receipt (measured when the child is 2 years of age), using multivariate probit models, controlling for age, race/ethnicity, educational attainment, prenatal health, and family history of depression. | (Noonan et al., 2016) |  |
| Child Outcomes | | | | | | | |  |
| MMHCs | Asthma | OR = 1.64 (1.25–2.17) | Pregnant women residing in the Avon area of southwest England | 1991–1999 | n = 5,810 | Longitudinal cohort study showing that maternal prenatal anxiety and depression at 32 weeks predicted greater child emotional and behavioral problems independent of a range of confounders (maternal age and education, crowding as index of socioeconomic status, birth weight and gestational age of the child, child sex, maternal prenatal smoking and substance use, maternal postnatal depression and anxiety, paternal pre- and postnatal anxiety, and a parenting index). | (Cookson et al., 2009) |  |
| MMHCs | Asthma | OR = 2.36 (1.61–3.45) | Nationally representative cohort of Australian children | 2004–2011 | n = 4,164 | Longitudinal cohort study using logistic regression analyses, controlling for risk factors, including child gender, maternal smoking during pregnancy, maternal use of asthma medication during pregnancy, instrumental delivery (cesarean, vacuum extraction, and/or forceps), preterm birth (< 37 weeks), low birth weight (< 2,500 grams), not being breastfed, attending a child care center within the first year of life, maternal age, number of children in the family, living in a metropolitan area, and socioeconomic status. | (Giallo et al., 2015) |  |
| MMHCs | Suboptimal breast-feeding | Any breastfeeding at 3 months: OR = 0.79 (0.70– 0.88) | Pregnancy Risk Assessment Monitoring System | 2010–2011 | n = 55,987 | Multivariable logistic regression was used to explore the association between a prepregnancy mental health visit and subsequent breastfeeding initiation, postpartum depression, and 3–month any and exclusive breastfeeding, controlling for adjusted for maternal race/ethnicity, age, marital status, pre–pregnancy mental health visit, and prenatal morbidity, abuse during or in the 12 months before pregnancy, and delivery type. | (Wouk et al., 2017) |  |
| Gestational diabetes mellitus | Cardio-vascular conditions | RR = 1.18 (1.11–1.25) | Women ages 18–45 with ascertainable diabetes status and continuous health plan enrollment at least 21 months before and 3 months after the birth | 2004–2011 | n = 839,792 | Retrospective claims analysis examining relative risk of costs and complications in pregnant patients with and without diabetes. | (Jovanovič et al., 2015) |  |
| MMHCs | Behavioral and develop-mental disorders | Probable child mental disorder: OR = 1.8 (1.62– 1.98) | Pregnant women residing in the Avon area of southwest England | Women who had an estimated date of delivery between April 1, 1991, and December 31, 1992 | n = 7,944 | Longitudinal cohort study showing that maternal prenatal anxiety and depression at 32 weeks predicted greater child emotional and behavioral problems independent of a range of confounders (maternal age and education, crowding as index of socioeconomic status, birth weight and gestational age of the child, child sex, maternal prenatal smoking and substance use, maternal postnatal depression and anxiety, paternal pre– and postnatal anxiety, and a parenting index). | (O’Donnell et al., 2014) |  |
| Hypertensive disorders | Behavioral and develop-mental disorders | Adjusted OR = 1.58 (1.334, 1.870) | Mother-child dyads of Medicaid births in South Carolina | Births in 1996–2002, followed through 2007 | n = 80,866 | Multiple logistic regression assessed the association between maternal preeclampsia and intellectual disability in the child. Authors controlled for maternal characteristics (race, age at delivery, high school graduation) and child characteristics (birth year, gender). | (Griffith et al., 2011) |  |
| Gestational diabetes mellitus | Behavioral and develop-mental disorders | Adjusted HR = 1.26 (1.14–1.41) | Singletons born in Kaiser Permanente Southern California hospitals | Births in 1995–2016, followed through age 4. | n = 333,182 | Retrospective birth cohort study that assessed the relative risk of ADHD associated with GDM using Cox regression models with adjustment for cofounders (including maternal age at delivery, parity, education, race/ethnicity, household income, maternal history of ADHD, history of medical comorbidity, birth year, and sex of the child). | (Xiang et al., 2018) |  |
| Gestational diabetes mellitus | Type 1 diabetes | RR = 1.13–4.36 | Multiple (review) | Studies published between 1963 and 2018 | 14 studies in the final review | Systematic review and meta-analysis of observational studies on the risk factors for child-onset type 1 diabetes mellitus. | (Hidayat et al., 2019) |  |
| MMHCs | ED visits | OR = 3.2 | Women in pediatric ED of University of Michigan health system | 2001–2002 | n = 176 | Cross-sectional study using multivariate regression models to examine the association of maternal depression in mothers of young children and child health care use, controlling for the presence of child chronic illness, child age, maternal age, years of maternal education, and insurance status. | (Flynn et al., 2004) |  |
| MMHCs | ED visits | Adjusted RR = 1.23 (1.12–1.34) | Kaiser Permanente membership system | 1997-2002 | n = 69,665 | Retrospective matched cohort design examining associations between parental depression and child health care use, controlling for child’s gender, number of parents, and a risk-adjustment variable to account for possible morbidity differences between exposed and unexposed children. We focused on adjusted rate ratios for children under 5 years of age. | (Sills et al., 2007) |  |
| Gestational diabetes mellitus | Fetal mal-formations | Adjusted OR = 1.32 (1.15–1.53) | All hospital births across Germany | 2006 | n = 668,085 | Authors conducted a cross-sectional study using the German Perinatal Quality Registry to assess the effects of GDM on the risk of adverse pregnancy outcomes. Multiple logistic regression models were used to estimate odds ratios. | (Schneider et al., 2011) |  |
| Gestational diabetes mellitus | Hypo-glycemia | Adjusted OR = 0.75 (0.65–0.87) | All infants born alive to Florida-resident women | 2004–2009 | n = 1,057,647 | Population-based retrospective cohort study. Authors linked a Florida statewide maternal and infant database with birth certificate records, death certificate records, and inpatient discharge records. Authors used logistic regression modeling to calculate odds ratios of the association between maternal GDM, body mass index (BMI), and adverse outcomes, controlling for maternal age, race/ethnicity, nativity, education, household income, parity, adequacy of prenatal care, infant sex, and tobacco, alcohol, and drug use during pregnancy. For our model, we used the OR for women with GDM and a normal BMI. | (Whiteman et al., 2015) |  |
| Gestational diabetes mellitus | Infection–related compli-cations | RR = 1.19 (1.10–1.27) | Women ages 18–45 with ascertainable diabetes status and continuous health plan enrollment at least 21 months before and 3 months after the birth | 2004–2011 | n = 839,792 | Retrospective claims analysis examining relative risk of costs and complications in pregnant patients with and without diabetes. | (Jovanovič et al., 2015) |  |
| MMHCs | Injury | Beta = 1.06 (0.58, 1.54) | Child Health and Human Development (NICHD) Study of Early Child Care, a longitudinal investigation of the effects of early child care on children's development | Not provided | n = 1,364 | Used Poisson models to assess maternal depression's effect on child injuries from birth to age 3, controlling for family socioeconomic background, child sex, child temperament and externalizing behavior, and parenting behaviors. | (Schwebel & Brezausek, 2008) |  |
| MMHCs | Injury | OR = 1.59 (1.24–2.04) | Japanese mothers and children | 2012 | n = 9,707 | Cross-sectional study using logistic multivariate regression to analyze the association between postpartum depression and experience of any unintentional injury, falls, and near drowning of infant children (4 months of age), adjusted for maternal characteristics (age, marital status, employment status, psychiatric history such as depression), paternal characteristics (age), infant characteristics (single or multiple birth, birth weight, gestational age, living with siblings), and household characteristics. | (Yamaoka et al., 2016) |  |
| MMHCs | Obesity | OR ranges from 0.28 (0.03–0.92) to 2.62 (1.02– 6.70) | Various (review) | Studies published between 2000 and 2014 | 2,033 records identified and 20 studies included in final review | Systematic review. Majority of studies showed positive associations between maternal depressive symptoms and increased risks for preschooler obesity. Effect sizes varied depending on the time at which depression was measured (i.e., antenatal, postnatal, in isolation, or longitudinally). | (Benton et al., 2015) |  |
| MMHCs | Obesity | Adjusted OR = 1.35 (1.06–1.72) | Fragile Families and Child Wellbeing Study | 2009 | n = 2,965 | Stepped multivariate analyses estimated the relationships between child obesity and maternal depression, adjusting for sociodemographic characteristics, child health and health behaviors, maternal health factors, and maternal–child relationship factors. | (Dow-Fleisner et al., 2021) |  |
| MMHCs | Obesity | Decreased chance of overweight (OR = 0.28, 95%; CI = 0.03 –0.92) | Latina mothers and their infants from 2 medical centers in San Francisco, CA (sample excluded women with drugs or alcohol abuse, diabetes, polycystic ovarian syndrome, eating disorders, or any health problems that would affect breastfeeding.) | 2006– 2007 | n = 181 | Longitudinal cohort study examining association between exposure to perinatal maternal depression and child weight-for length z-score at 6,12, and 24 months, controlling for infant birth weight, breastfeeding status, maternal postnatal BMI, maternal ethnicity, maternal age and gestational age. | (Wojcicki et al., 2011) |  |
| Hypertensive disorders | Poor fetal growth | 74.6 per 1,000 deliveries among women with preeclampsia; 25.9 per 1,000 deliveries among women without preeclampsia | National Inpatient Sample | 2014 | n = 3,796,490 | Authors used data from the Healthcare Cost and Utilization Project (HCUP) National Inpatient Sample (NIS) to characterize delivery hospitalizations involving preeclampsia/eclampsia. | (Fingar et al., 2017) |  |
| Gestational diabetes mellitus | Preterm birth | Adjusted OR = 1.27 (1.21–1.31) | Women ages 15–45 with singleton births in Catalonia with Caesarean and vaginal deliveries | 2011–2015 | n = 35,729 | Large population study based on a Spanish national database. Authors used multivariate and Poisson regression models to assess trends in GDM prevalence, standardized by maternal age. | (Gortazar et al., 2019) |  |
| Gestational diabetes mellitus | Preterm birth | Adjusted OR = 1.32 (1.25–1.39) | All infants born alive to Florida-resident women | 2004–2009 | n = 1,057,647 | Population-based retrospective cohort study. Authors linked a Florida statewide maternal and infant database with birth certificate records, death certificate records, and inpatient discharge records. Authors used logistic regression modeling to calculate odds ratios of the association between maternal GDM, BMI, and adverse outcomes, controlling for maternal age, race/ethnicity, nativity, education, household income, parity, adequacy of prenatal care, infant sex, and tobacco, alcohol, and drug use during pregnancy. For our model, we used the OR for women with GDM and a normal BMI. | (Whiteman et al., 2015) |  |
| Hemorrhage | Preterm birth | Hemorrhagic abnormally invasive placenta: RR = 3.2 (2.8–3.8)  Hemorrhagic abnormally invasive placenta: OR = 7.72 (5.82–10.2)  Abnormal bleeding of unknown origin: Adjusted OR = 4.31 (3.84–4.84)  Postpartum hemorrhage: OR = 1.51 (0.89-2.57)  Antepartum hemorrhage: OR = 2.81 (2.48–3.18)   Uterine rupture: Adjusted OR = 0.34 (0.08–1.45) | 6 studies from high-income countries (defined by the World Bank) | Studies published between 1994 and 2018 | 35 studies included in the final review | Systematic review/meta-analysis registered with the International Prospective Register of Systematic Reviews (PROSPERO) to detail the impact of severe maternal morbidity on perinatal outcomes in high-income countries. | (Mengistu et al., 2020) |  |
| Hypertensive disorders | Preterm birth | Severe gestational hypertension: OR = 7.18 (4.21–12.25)  Eclampsia: Adjusted OR = 4.808 (4.330–5.338) | 2 studies from high-income countries (defined by the World Bank) | Studies published between 1994 and 2018 | 35 studies included in the final review | Systematic review/meta-analysis registered with the International Prospective Register of Systematic Reviews (PROSPERO) to detail the impact of severe maternal morbidity on perinatal outcomes in high–income countries. | (Mengistu et al., 2020) |  |
| MMHCs | Preterm birth | ORs ranged from 1.16 (0.83–1.62) to 4.05 (1.38–11.87) | Varies (review) | Studies published in 2003 or later | 8 studies included in the final review | Meta-analysis of cohort studies with a follow-up of at least one month examining the adverse birth outcomes of pregnant women with any stress type. | (Lima et al., 2018) |  |
| MMHCs | Preterm birth | Adjusted RR = 1.49 (1.41–1.58) | Population‐ based administrative data holdings in Alberta, Canada, covering > 99.0% of the general province population | 2012–2015 | n = 158,486 | Multivariable log‐binomial regression models were used to assess the risk of adverse outcomes associated with depression alone (compared to without depression), adjusting for age and parity. | (Adhikari et al., 2019) |  |
| MMHCs | Preterm birth | Adjusted RR = 1.19 (1.16–1.22) | Nationwide Inpatient Sample | 2008–2014 | n = 5,518,766 | Multivariable logistic regressions adjusted for covariates: race, maternal age, insurance coverage (private insurance, Medicaid, or other), urban/rural hospital location, U.S. region, weekend, emergency department or elective admission, income, and admission year and other SMI diagnosis (MDD, BD, or schizophrenia). | (Heun-Johnson et al., 2019) |  |
| MMHCs | Preterm birth | Among high-quality U.S. studies, ORs for preterm birth ranged from OR = 0.71 (0.47–1.07) to OR = 4.97 (1.54–16.05) | Varies (review) | Studies published in 2010 or later | 23 studies included in the final review | Systematic review examining randomized and nonrandomized studies reporting the risk of adverse neonatal outcomes in pregnant women with untreated depression compared with pregnant women without depression. | (Jarde et al., 2016) |  |
| Gestational diabetes mellitus | Severe neonatal respiratory distress syndrome | Adjusted OR = 3.6 (1.5–8.6) | Women who delivered in the tertiary care-level maternity unit in Marseille | 2011–2012 | n = 444 | Prospective cohort study using a Chi squared, Fisher exact test, and Student t test to identify risk factors for respiratory distress syndrome. | (Mortier et al., 2017) |  |
| MMHCs | SIDS | OR = 4.93 (1.10-22.05) | Female patients registered in the UK General Practice Research Database | 1987–2000 | n = 169 cases; n = 662 controls | Case-control study of women with a live birth and subsequent SIDS death, compared to women with a live birth born the same year as the matched SIDS death, with infant survival for the first year of life. | (Howard et al., 2007) |  |
| MMHCs | SIDS | OR = 3.20 (1.46–6.99) | All births registered in Sheffield, UK | 1988–1993 | n = 32,984 | Case-control study comparing the rate of SIDS among mothers who had a high Edinburgh Postnatal Depression Scale (EPDS) score versus those who did not, controlling for mothers’ smoking status, residence in an area of poverty, preterm birth, maternal age, number of previous pregnancies, birth weight, number born (multiple births), maternal psychiatric history, year of birth (within study), month or season of birth, number of health visitor visits, baby’s sex, mother’s feeding intention (breast or bottle), mode of feeding at 1 month (breast or bottle), or maternal satisfaction with the infant’s feeding. | (Sanderson et al., 2002) |  |
| Cardio-vascular conditions | Stillbirth | The OR of stillbirth resulting from cardiac arrest = 14.84 (10.97–20.07) | 3 studies from high-income countries (defined by the World Bank) | Studies published between 1994 and 2018 | 35 studies included in the final review | Systematic review/meta-analysis registered with the PROSPERO to detail the impact of severe maternal morbidity on perinatal outcomes in high-income countries. | (Mengistu et al., 2020) |  |
| Hypertensive disorders | Stillbirth | HR = 1.6 (95% CI, 1.1–3.8) | Pregnant women and their babies enrolled in the Newborn Cross‐Sectional Study of the INTERGROWTH‐21st Project. | 2009–2013 | n = 60,121 | Large, multi‐country, multi‐ethnic, population‐based study; study populations were selected at the cluster-level (defined as urban areas where most women accessed antenatal care and delivered in medical facilities). Authors used a hierarchical approach for confounder selection and developed positive and negative likelihood ratios to identify plausible risk-factor combinations. | (Hirst et al., 2018) | |
| MMHCs | Attended well-child visit | OR = 0.81 (0.67–0.95) | National Evaluation of Healthy Steps for Young Children (HS) | 1996–1998 | n = 4,896 | Logistic regression for dichotomous outcomes and Poisson regression for count outcomes were used to estimate the effect of maternal depressive symptoms on children’s receipt of care. Models were adjusted for baseline demographic characteristics, child health status, and other potential confounders. | (Minkovitz et al., 2005) |  |

Notes: ED = emergency department; GDM = gestational diabetes mellitus; HR = hazard ratio; MMHCs = maternal mental health conditions; OECD = Organisation for Economic Co-operation and Development; OR = odds ratio; RR = risk ratio; SIDS = sudden infant death syndrome; SNAP = Supplemental Nutrition Assistance Program; TANF = Temporary Assistance for Needy Families; WCC = well-child care; WIC = Special Supplemental Nutrition Program for Women, Infants, and Children.
