# Supplementary material for: Societal cost of nine selected maternal morbidities in the United States
Source: PLoS One. 2022 Oct 26;17(10):e0275656. doi: 10.1371/journal.pone.0275656 (PMC9603953; doi:10.1371/journal.pone.0275656)
Supplement: S5 Appendix — (DOCX) [file pone.0275656.s005.docx]

# S5 Appendix. Model Inputs: Parameters and Costs Used to Estimate the Economic Impact of Maternal Morbidity Conditions Among 2019 Births

S5 Table 1. Parameters and costs: All U.S. births

| Parameter | Point estimate  (Range for sensitivity analyses) | Source |
| --- | --- | --- |
| Baseline demographic characteristics | | |
| Number of births | 3,745,540 | (Hamilton et al., 2020) |
| Number of pregnancies | 6,348,913 | (Curtin et al., 2015) |
| Prevalence of amniotic fluid embolism (%) | 0.0055 | (Knight et al., 2012) |
| Prevalence of cardiac arrest (%) | 0.0085 (0.0077–0.0093) | (Mhyre et al., 2014) |
| Prevalence of GDM (%) | 6.9 | (Martin et al., 2021) |
| Prevalence of hemorrhage (%) | 2.3 (1.1–3.4) | (Stranges et al., 2012) |
| Prevalence of hypertensive disorders (%) | 6.6 | (Butwick et al., 2020) |
| Prevalence of MMHCs (%) | 13.2 (12.6–13.8) | (Bauman et al., 2020) |
| Prevalence of renal disease (%) | 0.39 (0.35–0.42) | (Law et al., 2015) |
| Prevalence of sepsis (%) | 0.10 (0.09–0.10) | (Acosta et al., 2013) |
| Prevalence of venous thromboembolism (%) | 0.20 (0.15–0.25) | (Ghaji et al., 2013) |
| Other inputs |  |  |
| Medical care inflation (%) | 4.53 | (U.S. Bureau of Labor Statistics, 2020) |
| Discount rate (%) | 3.00 | (Sanders et al., 2016) |
| Women who do not achieve remission without treatment by the end of the first year postpartum (%) | 33.3 (20.0–60.0) | (Vliegen et al., 2014) |
| Maternal outcomes | | |
| **Cesarean section delivery** |  |  |
| Baseline incidence of cesarean section delivery (%) | 31.7 | (Hamilton et al., 2020) |
| Medical incremental costs per case of cesarean section delivery ($) | 12,179 | (Corry et al., 2013) |
| Likelihood of cesarean section delivery among birthing persons with GDM (%) | 43.3 (42.8–43.7) | (Jovanovič et al., 2015) |
| Likelihood of cesarean section delivery among birthing persons with MMHCs (%) | 40.5 (38.3–42.5) | (Bansil et al., 2010; Ogunyemi et al., 2018; Paul et al., 2013) |
| **Maternal mortality** |  |  |
| Baseline incidence of maternal mortality among birthing persons (%) | 0.020 | (Hoyert, 2021) |
| Medical incremental costs per case of cesarean section delivery ($) | 3,406 | (Shepard et al., 2016) |
| Nonmedical incremental costs per case of cesarean section delivery ($) | 42,900 | (U.S. Bureau of Labor Statistics, 2020) |
| Case fatality rate among birthing persons with amniotic fluid embolism (%) | 27.0 (11.0–43.0) | (Knight et al., 2012) |
| Case fatality rate among birthing persons with cardiac arrest (%) | 42.5 (30.0–55.0) | (Beckett et al., 2017) |
| Risk ratio of maternal mortality among birthing persons with hypertensive disorders | 1.82 (1.08–2.56) | (Ray et al., 2018) |
| Case fatality rate among birthing persons with renal disease (%) | 0.278 (0.138–0.418) | (Ray et al., 2018) |
| Rate of maternal mortality among birthing persons with sepsis (%) | 0.00113 (0.00077–0.00167) | (Cordioli et al., 2013) |
| Case fatality rate among birthing persons with venous thromboembolism (%) | 1.115 (0.320–1.910) | (Kourlaba et al., 2016) |
| **Peripartum stay** |  |  |
| Average peripartum stay (days) | 2.6 | (CMS, n.d.) |
| Medical daily cost per inpatient stay ($) | 2,723 | (AHRQ, n.d.) |
| Average peripartum stay for birthing persons with MMHCs (days) | 2.86 (2.64–3.08) | (Lancaster et al., 2010) |
| **Productivity loss** |  |  |
| Labor force participation among birthing persons with children aged younger than 6 years (%) | 63.8 | (U.S. Bureau of Labor Statistics, 2021) |
| Nonmedical per capita expected cost of job absenteeism ($) | 1,104 (415–1,792) | (Ammerman et al., 2016; Evans-Lacko and Knapp, 2016; Greenberg et al., 2015; Rost et al., 2004) |
| Nonmedical per capita expected cost of job presenteeism ($) | 3,107 (410–5,804) | (Evans-Lacko and Knapp, 2016; Greenberg et al., 2015; Rost et al., 2004) |
| Baseline rate of unemployment (%) | 4.0 | (U.S. Bureau of Labor Statistics, 2021) |
| Likelihood of unemployment among birthing persons with MMHCs (%) | 5.2 (4.8–5.6) | (Lerner and Henke, 2008) |
| Nonmedical cost per unemployed woman ($) | 42,900 | (U.S. Bureau of Labor Statistics, 2020) |
| **Social service use** |  |  |
| Baseline rate of SNAP receipt rate among women (%) | 46.7 | (U.S. Census Bureau, 2021a) |
| Likelihood of SNAP receipt among birthing persons with MMHCs (%) | 46.93 (46.84–47.02) | (Noonan et al., 2016) |
| Nonmedical cost per person receiving SNAP benefits ($) | 1,691 | (U.S. Food and Drug Administration, 2017) |
| Baseline rate of WIC receipt among women (%) | 26.3 | (U.S. Department of Agriculture, 2020) |
| Likelihood of WIC receipt among women with MMHCs (%) | 26.48 (26.39–26.56) | (Noonan et al., 2016) |
| Nonmedical cost per person receiving WIC benefits ($) | 816 | (U.S. Department of Agriculture, 2019a, 2019d, 2019b) |
| Baseline rate of Medicaid receipt women (%) | 42.1 | (Martin et al., 2020) |
| Likelihood of Medicaid receipt among women with MMHCs (%) | 42.35 (42.25–42.44) | (Noonan et al., 2016) |
| Nonmedical cost per person receiving Medicaid benefits ($) | 7,883 | (Centers for Medicare and Medicaid Services, 2017) |
| Baseline rate of TANF receipt among women (%) | 2.5 | (U.S. Census Bureau, 2021b; U.S. Department of Health and Human Services, 2020) |
| Likelihood of TANF receipt among women with MMHCs (%) | 2.70 (2.59–2.81) | (Noonan et al., 2016) |
| Nonmedical per person receiving TANF benefits ($) | 10,374 | (U.S. Department of Health and Human Services, 2018; U.S. Department of Health and Human Services, 2017) |
| **Stroke** |  |  |
| Baseline incidence (%) | 0.0058 | (Miller et al., 2020) |
| Medical incremental cost per case of stroke ($) | 20,719 | (AHRQ, n.d.) |
| Likelihood of stroke among women with hypertensive disorders (%) | 0.024 (0.014–0.034) | (Wu et al., 2017) |
| **Suicide** |  |  |
| Baseline incidence (%) | 0.0064 | (United Health Foundation, 2020) |
| Medical incremental cost per case of suicide ($) | 3,406 | (Shepard et al., 2016) |
| Nonmedical incremental cost per case of suicide ($) | 42,900 | (U.S. Bureau of Labor Statistics, 2020) |
| Likelihood of suicide among women with depression (%) | 0.1504 (0.1280–0.1728) | (Ösby et al., 2001) |
| Child outcomes | | |
| **Asthma** |  |  |
| Baseline incidences (%) | 2.6 | (CDC, 2020d) |
| Medical incremental cost per child with asthma ($) | 955 | (Sullivan et al., 2017) |
| Nonmedical incremental cost per child with asthma ($) | 425 | (Nurmagambetov et al., 2017) |
| Likelihood of asthma among children born to persons with MMHCs (%) | 5.1 (4.2–5.9) | (Cookson et al., 2009; Giallo et al., 2015) |
| **Breastfeeding** |  |  |
| Baseline incidence (%) | 46.9 | (CDC, 2020c) |
| Medical incremental cost per infant due to suboptimal breastfeeding ($) | 2,652 | (Bartick and Reinhold, 2010) |
| Likelihood of breastfeeding among children born to persons with MMHCs (%) | 41.1 (38.2–43.7) | (Wouk et al., 2017) |
| **Cardiovascular conditions** |  |  |
| Baseline incidence (%) | 0.20 | (Mai et al., 2019) |
| Medical incremental cost per child with cardiovascular conditions ($) | 58,842 | (Faraoni et al., 2016) |
| Likelihood of cardiovascular conditions among children born to persons with GDM (%) | 0.24 (0.22–0.25) | (Jovanovič et al., 2015) |
| **Child behavioral and developmental disorders** |  |  |
| Baseline incidence (%) | 17.3 | (Cree et al., 2018) |
| Nonmedical incremental cost per child with a behavioral or developmental disorder ($) | 12,990 | (Kancherla et al., 2012) |
| Likelihood of behavioral or developmental disorders among children born to persons with GDM (%) | 21.7 (19.7–23.7) | (Xiang et al., 2018) |
| Likelihood of behavioral or developmental disorders among children born to persons with hypertensive disorders (%) | 25.2 (21.9–28.3) | (Griffith et al., 2011) |
| Likelihood of behavioral or developmental disorders among children born to persons with MMHCs (%) | 27.5 (25.4–29.4) | (O’Donnell et al., 2014) |
| **Diabetes** |  |  |
| Baseline incidence (%) | 0.022 | (Divers et al., 2020) |
| Medical incremental cost per child with diabetes ($) | 15,649 | (Lee et al., 2015) |
| Likelihood of behavioral or developmental disorders among children born to persons with GDM (%) | 0.060 (0.025–0.096) | (Hidayat et al., 2019) |
| **Emergency department (ED) visits** |  |  |
| Baseline incidence (%) | 58.4 | (Cairns et al., 2018) |
| Medical incremental cost per child with ED visits ($) | 805 | (AHRQ, 2019) |
| Likelihood of ED visit among children born to persons with MMHCs (%) | 75.7 (63.3–81.8) | (Flynn et al., 2004; Sills et al., 2007) |
| **Fetal malformations** |  |  |
| Baseline incidence (%) | 26.2 | (Law et al., 2015) |
| Medical incremental cost per child with fetal malformations ($) | 64,153 | (Faraoni et al., 2016) |
| Likelihood of fetal malformations among children born to persons with GDM (%) | 32.2 (29.0–35.2) | (Schneider et al., 2011) |
| **Hypoglycemia** |  |  |
| Baseline incidence (%) | 10.0 | (Thompson-Branch and Havranek, 2017) |
| Medical incremental cost per child with hypoglycemia ($) | 20,500 | (AHRQ, n.d.) |
| Likelihood of hypoglycemia among children born to persons with GDM (%) | 7.7 (6.7–8.6) | (Whiteman et al., 2015) |
| **Infection** |  |  |
| Baseline incidence (%) | 0.74 | (Fleischmann-Struzek et al., 2018) |
| Medical incremental cost per child with infection ($) | 10,059 | (AHRQ, n.d.) |
| Likelihood of infection among children born to persons with GDM (%) | 0.88 (0.81-0.94) | (Jovanovič et al., 2015) |
| **Injury** |  |  |
| Baseline incidence (%) | 7.9 | (CDC, 2020b) |
| Medical incremental cost per child with an injury ($) | 8,018 | (CDC, 2019) |
| Likelihood of injury among children born to persons with MMHCs (%) | 10.2 (8.3–12.0) | (Schwebel & Brezausek, 2008; Yamaoka et al., 2016) |
| **Obesity** |  |  |
| Baseline incidence (%) | 13.4 | (Fryar et al., 2020) |
| Medical incremental cost per child with obesity ($) | 200 | (Finkelstein et al., 2014) |
| Likelihood of obesity among children born to persons with MMHCs (%) | 18.3 (4.2–28.8) | (Benton et al., 2015; Dow-Fleisner et al., 2021; Wojcicki et al., 2011) |
| **Poor fetal growth** |  |  |
| Baseline incidence (%) | 2.6 | (Fingar et al., 2017) |
| Medical incremental cost per child with poor fetal growth ($) | 8,562 | (AHRQ, n.d.) |
| Likelihood of poor fetal growth for deliveries to persons with hypertensive disorders (%) | 7.5 | (Fingar et al., 2017) |
| **Preterm birth** |  |  |
| Baseline incidence (%) | 10.2 | (Martin et al., 2021) |
| Medical incremental cost per child with preterm birth ($) | 78,052 | (Hall & Greenberg, 2016) |
| Likelihood of preterm birth among infants born to persons with GDM (%) | 12.8 (12.6–13.0) | (Gortazar et al., 2019; Whiteman et al., 2015) |
| Likelihood of preterm birth among infants born to persons with hemorrhage (%) | 37.4 (24.2–46.7) | (Mengistu et al., 2020) |
| Likelihood of preterm birth among infants born to persons with hypertensive disorders (%) | 40.5 (35.3–44.9) | (Mengistu et al., 2020) |
| Likelihood of preterm birth among infants born to persons with MMHCs (%) | 24.4 (7.5–36.1) | (Adhikari et al., 2019; Heun-Johnson et al., 2019; Jarde et al., 2016; Lima et al., 2018) |
| **Respiratory distress syndrome** |  |  |
| Baseline incidence (%) | 3.3 | (Khemani et al., 2019) |
| Medical incremental cost per case of respiratory distress syndrome ($) | 72,335 | (AHRQ, n.d.) |
| Likelihood of respiratory distress syndrome among children born to persons with GDM (%) | 14.7 (4.9–22.7) | (Mortier et al., 2017) |
| **SIDS** |  |  |
| Baseline incidence (%) | 0.035 | (CDC, 2020a) |
| Nonmedical incremental cost per case of SIDS ($) | 23,691 | (Fox et al., 2014) |
| Likelihood of SIDS among children born to persons with MMHCs (%) | 0.142 (0.112–0.173) | (Howard et al., 2007; Sanderson et al., 2002) |
| **Stillbirth** |  |  |
| Baseline incidence (%) | 0.59 | (Hoyert and Gregory, 2020) |
| Medical incremental cost per stillbirth ($) | 9,611 | (Heazell et al., 2016) |
| Nonmedical incremental cost per stillbirth ($) | 3,448 | (Heazell et al., 2016) |
| Likelihood of stillbirth for pregnancies to persons with cardiac arrest (%) | 8.39 (6.08–10.59) | (Mengistu et al., 2020) |
| Likelihood of stillbirth for pregnancies to birthing persons with hypertensive disorders (%) | 1.42 (0.65–2.17) | (Hirst et al., 2018) |
| **Attending WCC visits** |  |  |
| Baseline likelihood of attending a WCC visit (%) | 65.0 | (Wolf et al., 2018) |
| Medical cost per WCC visit ($) | 181 | (AHRQ, 2015) |
| Likelihood of attending WCC visit among children born to persons with MMHCs (%) | 60.1 (55.4–63.8) | (Minkovitz et al., 2005) |

Notes: ED = emergency department; GDM = gestational diabetes mellitus; MMHCs = maternal mental health conditions; SIDS = sudden infant death syndrome; SNAP = Supplemental Nutrition Assistance Program; TANF = Temporary Assistance for Needy Families; WCC = well-child care; WIC = Special Supplemental Nutrition Program for Women, Infants, and Children.
