# Supplementary material for: Societal cost of nine selected maternal morbidities in the United States
Source: PLoS One. 2022 Oct 26;17(10):e0275656. doi: 10.1371/journal.pone.0275656 (PMC9603953; doi:10.1371/journal.pone.0275656)
Supplement: S6 Appendix — (DOCX) [file pone.0275656.s006.docx]

# S6 Appendix. Modeling Method

Our study estimated the societal cost of maternal morbidity by calculating the excess cost of outcomes directly attributable to each maternal morbidity. After a comprehensive literature review, we identified connections between nine maternal morbidity conditions and 24 maternal and child outcomes that had (1) strong support in the literature, and (2) associated medical and/or nonmedical costs readily available (i.e., without any additional primary data analysis). We created a macro-enabled Excel workbook that combined the baseline rates of each condition and outcome, impact estimates for each connection, and associated costs to estimate the total societal cost of maternal morbidity from conception through five years postpartum.

We calculated the annual incremental excess cost of each outcome due to each maternal morbidity condition. In our model workbook, we calculated the societal costs associated with the incremental number of persons (either birthing people or children) who would experience each outcome directly due to a maternal morbidity condition. Table F.1 delineates the steps needed to calculate the excess costs of each morbidity-outcome connection.

**S6 Table 1. Formulas used to calculate the annual incremental excess cost of each outcome**

| **Step** | **Formula** |
| --- | --- |
| 1 | $Baseline expected number of people with outcome=baseline occurrence of outcome\times population$ |
| 2 | $Likelihood of outcome given condition=baseline occurrence of outcome\times impact estimate$ |
| 3 | $Expected number of people with outcome who have condition=likelihood of outcome given condition\times population who have condition$ |
| 4 | $Expected number of people with outcome who do not have condition=baseline occurrence of outcome\times population who do not have condition$ |
| 5 | $Expected number of people with outcome weighted for condition occurrence=Expected number of people with outcome who have condition (Step 3)+ Expected number of people with outcome who do not have condition (Step 4)$ |
| 6 | $Excess number of people with outcome due to condition =Expected number of people with outcome weighted for condition occurrence (Step 5)- Baseline expected number of people with outcome (Step 1)$ |
| 7 | $Excess cost due to condition=Excess number of people with outcome due to condition (Step 6) \times cost of outcome$ |

For example, the annual incremental excess cost estimate for the impact of gestational diabetes mellitus (GDM) on cesarean section deliveries represents the incremental risk of a birthing person with GDM needing a cesarean section compared to a birthing person without GDM.

The following rules for rounding estimates were used: prevalence estimates are rounded to three decimal places with a minimum of two significant digits; odds ratios are rounded to the number of decimal places to which the prevalence is rounded (maximum four decimal places); impact estimates are rounded to four decimal places; costs and numbers of births/pregnancies are rounded to the nearest whole number.

To project costs through five years postpartum, we assumed that:

1. Increased rates of cesarean section delivery, maternal mortality, peripartum stays, stroke, suboptimal breastfeeding, fetal malformations, hypoglycemia, infection in child, poor fetal growth, preterm birth, respiratory distress in child, SIDS, and stillbirth occur only in the first year of the model.
2. The costs of productivity loss, suicide, increased incidence of child injury, increased incidence of emergency department visits, and decreased likelihood of attending annual well-child care visits occur annually until the birthing persons achieves remission from the maternal morbidity. When the birthing person achieves remission, costs of these outcomes due to maternal morbidity fall to $0.
3. Roughly two-thirds of birthing people achieve remission from MMHCs by the end of the first year of the model, even without treatment. Following the meta-analysis by Vliegen et al. (2014), we assumed that the proportion achieving remission by the end of each year remains constant across years.
4. The impact of excess maternal morbidity conditions on the outcomes of social service use, child asthma, cardiovascular conditions, behavioral and developmental disorders, diabetes, and obesity, remains constant over time. For example, we assumed that a child with a behavioral or developmental disorder would continue to have that disorder even if their respective birthing person recovers from the maternal morbidity within five years postpartum.

We discounted costs at an annual rate of 3% to reflect the lower present economic value of an expense occurring in the future (Sanders et al., 2016). Finally, to project costs through five years postpartum, we adjusted for inflation using the medical component of the CPI. Based on the percentage change between the December 2018 medical CPI and the December 2019 medical CPI, we assumed that medical costs increased by 4.53% each year after Year 0 (U.S. Bureau of Labor Statistics, 2020). We collated estimates by condition and developed the model in Microsoft Excel, using Visual Basic to develop macros that update the model when inputs are changed (Office 365, Microsoft, Redmond, WA).
