# Supplementary material for: Societal cost of nine selected maternal morbidities in the United States
Source: PLoS One. 2022 Oct 26;17(10):e0275656. doi: 10.1371/journal.pone.0275656 (PMC9603953; doi:10.1371/journal.pone.0275656)
Supplement: S7 Appendix — (DOCX) [file pone.0275656.s007.docx]

# S7 Appendix. Sensitivity Analyses

To determine which model parameters had the greatest impact on estimated costs, we conducted sensitivity analyses. Where possible, we varied the prevalence of maternal morbidity conditions, the rate of remission from MMHCs, and impact estimates, because these parameters had a higher degree of uncertainty. For many parameters, we could not find multiple values and/or additional values did not meet the inclusion criteria; we could only conduct sensitivity analyses on parameters with multiple values. Additionally, we did not vary baseline rates of outcomes because of greater certainty about these rates based on the literature. S5 Table 1 shows the range of input parameters used in the sensitivity analyses.

The tornado diagram in S7 Figure 1 shows the results of the sensitivity analyses, including the difference in costs, in billions of dollars, from the main model. Each parameter varied from its lowest to its highest value. Overall, varying impact estimates resulted in larger changes to the cost estimates than did varying the prevalence of maternal morbidity conditions and varying the remission rate for MMHCs. Varying all impact estimates at once resulted in our estimate with a range of total societal costs over five years from $13.6 billion to $48.6 billion dollars (or $18.7 billion lower to $16.3 billion higher than our main estimate); varying all prevalence estimates at once resulted in a $1.8 billion lower estimate and a $1.7 billion higher estimate; and varying the remission rate for MMHCs resulted in a $1.8 billion lower and a $3.7 billion higher estimate.

**S7 Figure 1. Tornado diagram**

**
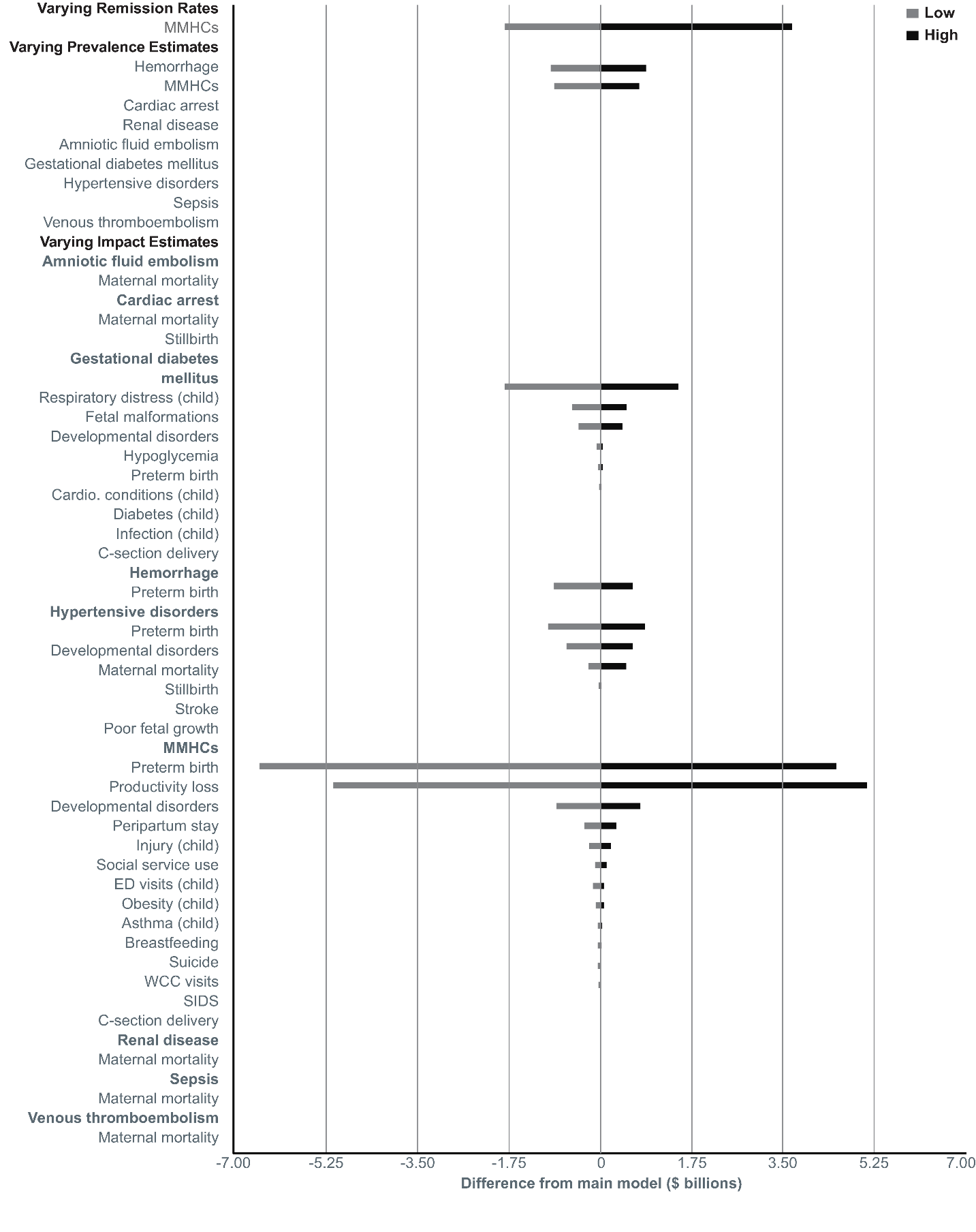
**

Notes: This figure shows the results of our sensitivity analyses, including the difference in costs, in billions of dollars, from the main model as we varied each parameter from its lowest to its highest value.

The parameters with the greatest effects on estimated costs included the impact estimate for exposure to MMHCs on preterm birth (from $6.5 billion lower to $4.5 billion higher), followed by the impact estimate for exposure to MMHCs on productivity loss (from $5.1 billion lower to $5.1 billion higher), and the remission rate for MMHCs (from $1.8 billion lower to $3.7 billion higher). These parameters all incorporate MMHCs, which have a high prevalence and large ranges in the literature. The associated costs were very high, so varying these parameters led to substantial variation in model results. Other parameters with a large range of values included a condition with a relatively low prevalence or associated costs, and therefore did not result in large variation in our model results. For example, amniotic fluid embolism had a large range of impact estimated values, but a low prevalence.

Among non-MMHC conditions, the parameters with the largest effects on our model results included the impact estimate for exposure to GDM on child respiratory distress syndrome (from $1.8 billion lower to $1.5 billion higher), the impact estimate of exposure to hypertensive disorders on preterm birth (from $1.0 billion lower to $0.8 billion higher), and the prevalence estimate of hemorrhage (from $1.0 billion lower to $0.9 billion higher).
