# Supplementary material for: Societal cost of nine selected maternal morbidities in the United States
Source: PLoS One. 2022 Oct 26;17(10):e0275656. doi: 10.1371/journal.pone.0275656 (PMC9603953; doi:10.1371/journal.pone.0275656)
Supplement: S8 Appendix — (DOCX) [file pone.0275656.s008.docx]

# S8 Appendix. Full Results of the Model

**S8 Table 1. Full results of the model, by condition and outcome**

| **Outcomes** | **Total** | **Year 0** | **Year 1** | **Year 2** | **Year 3** | **Year 4** | **Year 5** |
| --- | --- | --- | --- | --- | --- | --- | --- |
| Amniotic fluid embolism |  |  |  |  |  |  |  |
| Maternal |  |  |  |  |  |  |  |
| Maternal mortality | 4.4 | 4.4 | 0.0 | 0.0 | 0.0 | 0.0 | 0.0 |
| **Total** | **4.4** | **4.4** | **0.0** | **0.0** | **0.0** | **0.0** | **0.0** |
| Cardiac arrest |  |  |  |  |  |  |  |
| Maternal |  |  |  |  |  |  |  |
| Maternal mortality | 10.6 | 10.6 | 0.0 | 0.0 | 0.0 | 0.0 | 0.0 |
| Child |  |  |  |  |  |  |  |
| Stillbirth | 0.3 | 0.3 | 0.0 | 0.0 | 0.0 | 0.0 | 0.0 |
| **Total** | **10.9** | **10.9** | **0.0** | **0.0** | **0.0** | **0.0** | **0.0** |
| Gestational diabetes mellitus |  |  |  |  |  |  |  |
| Maternal |  |  |  |  |  |  |  |
| C-section delivery | 365.1 | 365.1 | 0.0 | 0.0 | 0.0 | 0.0 | 0.0 |
| Child |  |  |  |  |  |  |  |
| Behavioral or developmental disorders | 899.2 | 144.4 | 150.9 | 157.8 | 164.9 | 172.4 | 180.2 |
| Cardiovascular conditions | 38.1 | 6.1 | 6.4 | 6.7 | 7.0 | 7.3 | 7.6 |
| Diabetes | 9.4 | 1.5 | 1.6 | 1.6 | 1.7 | 1.8 | 1.9 |
| Fetal malformations | 994.8 | 994.8 | 0.0 | 0.0 | 0.0 | 0.0 | 0.0 |
| Hypoglycemia | -121.9 | -121.9 | 0.0 | 0.0 | 0.0 | 0.0 | 0.0 |
| Infection | 3.6 | 3.6 | 0.0 | 0.0 | 0.0 | 0.0 | 0.0 |
| Preterm birth | 524.5 | 524.5 | 0.0 | 0.0 | 0.0 | 0.0 | 0.0 |
| Respiratory distress syndrome/failure | 2,131.1 | 2,131.1 | 0.0 | 0.0 | 0.0 | 0.0 | 0.0 |
| **Total** | **4,843.9** | **4,049.2** | **158.9** | **166.1** | **173.6** | **181.5** | **189.7** |
| Hemorrhage |  |  |  |  |  |  |  |
| Child |  |  |  |  |  |  |  |
| Preterm birth | 1,828.9 | 1,828.9 | 0.0 | 0.0 | 0.0 | 0.0 | 0.0 |
| **Total** | **1,828.9** | **1,828.9** | **0.0** | **0.0** | **0.0** | **0.0** | **0.0** |
| Hypertensive disorders |  |  |  |  |  |  |  |
| Maternal |  |  |  |  |  |  |  |
| Maternal mortality | 3.1 | 3.1 | 0.0 | 0.0 | 0.0 | 0.0 | 0.0 |
| Stroke | 1.0 | 1.0 | 0.0 | 0.0 | 0.0 | 0.0 | 0.0 |
| Child |  |  |  |  |  |  |  |
| Behavioral or developmental disorders | 1,559.9 | 250.5 | 261.8 | 273.7 | 286.1 | 299.1 | 312.6 |
| Poor fetal growth | 103.7 | 103.7 | 0.0 | 0.0 | 0.0 | 0.0 | 0.0 |
| Preterm birth | 5,846.3 | 5,846.3 | 0.0 | 0.0 | 0.0 | 0.0 | 0.0 |
| Stillbirth | 26.8 | 26.8 | 0.0 | 0.0 | 0.0 | 0.0 | 0.0 |
| **Total** | **7,540.8** | **6,231.4** | **261.8** | **273.7** | **286.1** | **299.1** | **312.6** |
| Maternal mental health conditions |  |  |  |  |  |  |  |
| Maternal |  |  |  |  |  |  |  |
| C-section delivery | 529.9 | 529.9 | 0.0 | 0.0 | 0.0 | 0.0 | 0.0 |
| Peripartum stay | 350.0 | 350.0 | 0.0 | 0.0 | 0.0 | 0.0 | 0.0 |
| Productivity loss | 6,615.8 | 2,413.9 | 840.2 | 878.3 | 918.1 | 959.7 | 1,003.2 |
| Social service use | 239.1 | 38.4 | 40.1 | 42.0 | 43.9 | 45.8 | 47.9 |
| Suicide | 153.2 | 55.9 | 19.5 | 20.3 | 21.3 | 22.2 | 23.2 |
| Child |  |  |  |  |  |  |  |
| Asthma | 106.4 | 17.1 | 17.9 | 18.7 | 19.5 | 20.4 | 21.3 |
| Attended well-child care visits | -12.0 | -4.4 | -1.5 | -1.6 | -1.7 | -1.7 | -1.8 |
| Behavioral or developmental disorders | 4,039.7 | 648.7 | 678.1 | 708.8 | 740.9 | 774.5 | 809.6 |
| Childhood obesity | 29.8 | 4.8 | 5.0 | 5.2 | 5.5 | 5.7 | 6.0 |
| Emergency department visits | 188.9 | 68.9 | 24.0 | 25.1 | 26.2 | 27.4 | 28.6 |
| Injury | 250.0 | 91.2 | 31.7 | 33.2 | 34.7 | 36.3 | 37.9 |
| Preterm birth | 5,479.7 | 5,479.7 | 0.0 | 0.0 | 0.0 | 0.0 | 0.0 |
| Sudden infant death syndrome | 12.5 | 12.5 | 0.0 | 0.0 | 0.0 | 0.0 | 0.0 |
| Suboptimal breastfeeding | 76.0 | 76.0 | 0.0 | 0.0 | 0.0 | 0.0 | 0.0 |
| **Total** | **18,059.0** | **9,782.6** | **1,655.0** | **1,730.0** | **1,808.4** | **1,890.3** | **1,975.9** |
| Renal disease |  |  |  |  |  |  |  |
| Maternal |  |  |  |  |  |  |  |
| Maternal mortality | 3.0 | 3.0 | 0.0 | 0.0 | 0.0 | 0.0 | 0.0 |
| **Total** | **3.0** | **3.0** | **0.0** | **0.0** | **0.0** | **0.0** | **0.0** |
| Sepsis |  |  |  |  |  |  |  |
| Maternal |  |  |  |  |  |  |  |
| Maternal mortality | 3.3 | 3.3 | 0.0 | 0.0 | 0.0 | 0.0 | 0.0 |
| **Total** | **3.3** | **3.3** | **0.0** | **0.0** | **0.0** | **0.0** | **0.0** |
| Venous thromboembolism |  |  |  |  |  |  |  |
| Maternal |  |  |  |  |  |  |  |
| Maternal mortality | 6.4 | 6.4 | 0.0 | 0.0 | 0.0 | 0.0 | 0.0 |
| **Total** | **6.4** | **6.4** | **0.0** | **0.0** | **0.0** | **0.0** | **0.0** |
| **Total societal costs for the 2019 birth cohort** | **32,301** | **21,920.1** | **2,075.7** | **2,169.8** | **2,268.1** | **2,370.9** | **2,478.2** |
