# Supplementary material for: Societal cost of nine selected maternal morbidities in the United States
Source: PLoS One. 2022 Oct 26;17(10):e0275656. doi: 10.1371/journal.pone.0275656 (PMC9603953; doi:10.1371/journal.pone.0275656)
Supplement: S9 Appendix — (DOCX) [file pone.0275656.s009.docx]

# S9 Appendix. Glossary

**Absenteeism.** An inability to work.

**Behavioral and developmental disorders.** A series of disorders, such as attention-deficit/hyperactivity disorder, depression, anxiety, and behavioral or conduct disorders such as oppositional defiant disorder, which occur in children and can lead to reduced educational attainment in the longer term.

**Cardiovascular conditions.** Conditions that affect the heart or blood vessels.

**Fetal malformations.** A physical defect present in a child at birth.

**Hypoglycemia.** A condition where glucose levels are lower than normal.

**Maternal mental health conditions.** Conditions that occur in the perinatal period, between conception and five years postpartum. Depression is the most common condition, but mothers may also develop anxiety disorders, which include generalized anxiety disorder, panic disorder, obsessive-compulsive disorder, or birth-related post-traumatic stress disorder.

**Medical Costs.** Costs incurred during the prevention or treatment of injury or disease borne by the health care delivery system. These costs include, but are not limited to prescriptions, outpatient costs, inpatient costs, and behavioral health costs.

**Nonmedical Costs.** All costs not incurred during the prevention or treatment of injury or disease. These costs include, but are not limited to lost wages, the cost of decreased productivity, educational costs, and funeral costs.

**Preeclampsia.** A potentially dangerous pregnancy complication involving high blood pressure, swelling of hands and feet, and protein in urine. If left untreated, preeclampsia can lead to eclampsia, which poses severe health risks for mother and child and may lead to death.

**Presenteeism.** Reduced productivity and an increased likelihood of making mistakes at work.

**Poor fetal growth.** When the fetus has not grown as large as would be expected for stage of the pregnancy.

**Racial and ethnic backgrounds.** These are defined according to the 1997 Office of Management and Budget (OMB) standards on race and ethnicity, which are used by the U.S. Census Bureau:

- **Hispanic.** A mother of Cuban, Mexican, Puerto Rican, South or Central American, or other Spanish heritage. They may be of any race.
- **Non-Hispanic White.** A non-Hispanic mother of European, Middle Eastern, or North African heritage.
- **Non-Hispanic Black.** A non-Hispanic mother of Black African heritage.

**Social Service Use.** Public assistance funding provided through the Supplemental Nutrition Assistance Program (SNAP), Special Supplemental Nutrition Program for Women, Infants, and Children (WIC), Medicaid, and Temporary Assistance for Needy Families (TANF).

**Suboptimal breastfeeding.** Not breastfeeding at all, or not exclusively breastfeeding through three months postpartum.
