# Supplementary material for: Societal cost of nine selected maternal morbidities in the United States
Source: PLoS One. 2022 Oct 26;17(10):e0275656. doi: 10.1371/journal.pone.0275656 (PMC9603953; doi:10.1371/journal.pone.0275656)
Supplement: Attachment — Submitted filename: Response to Reviewers.docx [file pone.0275656.s010.docx]

Sasigant So O’Neil, SM

Mathematica, P.O. Box 2393

Princeton, NJ 08543-2393

(617) 301-8975

[soneil@mathematica-mpr.com](mailto:soneil@mathematica-mpr.com)

Emily Chenette, PhD

Editor-in-Chief, PLOS ONE

1265 Battery Street, Suite 200

San Francisco, CA 94111, USA

June 17, 2022

Dear Dr. Chenette,

My co-authors and I were pleased to receive your response on May 18, 2022 inviting us to revise and resubmit our manuscript. Accordingly, we submit the enclosed revised paper, “Societal Cost of Nine Selected Maternal Morbidities in the United States,” Manuscript # PONE-D-22-11649, for publication in *PLOS ONE*.

We thank you and the reviewers for your time and effort in reviewing our manuscript. The feedback has been invaluable in improving the content and presentation of the paper.

We revised our manuscript according to reviewer comments and addressed them directly in the table accompanying this letter. Our revised submission includes (1) a revised manuscript with track changes visible under the All Markup mode of Microsoft Word, (2) a clean version of the manuscript, and (3) a clean version of Appendix S4 with an updated title (title changes provided in track in the manuscript).

All authors have read and approved the revised manuscript. We hope that our resubmission is now suitable for inclusion in *PLOS ONE* and we look forward to hearing from you.

Sincerely,

Sasigant So O’Neil, SM

Director, Domestic Health Philanthropy Portfolio

Mathematica

| Reviewer #1 | Author Response |
| --- | --- |
| Thank you for the opportunity to review this critically important and timely study. O’Neil et al. address an important policy and public health issue – societal costs of maternal morbidity. The paper is well written and applies appropriate methods to advance the topic. I have several suggestions and requests for points of clarification that I believe will help to strengthen the paper. These concerns and minor feedback are detailed below, by section: | We thank the reviewer for this comment. |
| **Introduction:** |  |
| Line 13 - I suggest replacing 'developed' with high-income or industrialized countries. | We replaced “developed countries” with “high-income countries.” |
| Line 25 - Is 2019 in the "maternal-pair from pregnancy to five years postpartum" referring to the end of postpartum period? It is confusing to read about the 2019 birth cohort and tracking the maternal-child pair 5 years postpartum (meaning to 2023?) - please clarify. | We thank the reviewer for pointing out this ambiguity. The analysis tracks the maternal and child costs for the 2019 birth cohort through 2023, 5 years postpartum. We clarified the text. |
| **Methods:** |  |
| Line 93 - Could you please include a reference/citation to the Healthcare Cost and Utilization Project from which the Nationwide Inpatient Sample was drawn for those readers not familiar with it? | We added a reference to the Healthcare Cost and Utilization Project Nationwide Inpatient Sample webpage. |
| Lines 126-128 - I am not convinced about the causal language used in key parameters 3 and 4 - does this mean you only selected those studies that estimated the impact (causal relationship)? Or how do you know the incidence of outcome (3) was caused by the maternal morbidity? Also, odds ratio estimates the association between morbidity-outcome, and not the impact of morbidity on outcome. I advise the authors to either clarify the criteria or adjust the language used to avoid inferences about the causality between morbidity-outcome. | For key parameter 3, we changed “resulting from” to “associated with” to remove causal language. Our model aims to estimate the costs of *excess risk* of outcomes. |
| **Results:** |  |
| Table 1 - please include a brief explanation as to why some maternal morbidity outcomes have cost estimates for 1 year and other for up to 5 years? | We updated the Table 1 legend to contain the following language that describes why some conditions contain costs for one year and others for five years: “Maternal morbidity conditions with acute outcomes have one-time costs, while conditions with chronic outcomes have ongoing costs, modeled through five years postpartum. Appendix S8 contains the full results of the model by condition and outcome.” |
| **Discussion:** |  |
| Lines 223-224 - I think the language like this has to be attenuated unless you only included those studies that looked at the causal effect and thus provide evidence that those outcomes truly resulted from / were caused by maternal morbidity? | We changed “resulting from” to “associated with” to remove causal language in this sentence and elsewhere in the manuscript. |
| Limitations - I appreciate the detailed discussion (and acknowledgment) of study limitations! | We thank the reviewer for this comment. |

| S4 Appendix |  |
| --- | --- |
| S4 Appendix. Effects of Exposure to Maternal Morbidity Conditions – Again, I would suggest adjusting the language implying causality between maternal morbidity conditions and maternal and child health outcomes (e.g. “Evidence of the association between MMC and maternal and child health” or “MCH outcomes associated with MMC” ) | We revised the title of this appendix to “Evidence of Association between Maternal Morbidity Conditions and Maternal and Child Outcomes” per the reviewer’s suggestion to remove causal language. |
| I am impressed with the authors’ rigorous and transparent approach to documenting the conduct of this study and providing so much supporting information in the appendices. This is very helpful and greatly appreciated. | We thank the reviewer for this comment. |

| Reviewer #2 | Author Response |
| --- | --- |
| This is a meaningful contribution to the body of literature about maternal morbidity. Akin to the referenced publication by Luca et all assigning a cost to the impact of MMHC, this manuscript attempts to estimate the cost of several maternal morbidities. This analysis addresses the question of how to assign economic metrics to maternal morbidity beyond the associated hospitalization and recognizes that these experiences have ramifications for maternal and early childhood health. | We thank the reviewer for this comment. |
| As an obstetric reader of this, I need a better explanation of why this list of 9 morbidities. AFE, Cardiac arrest, AKI, sepsis, VTE are all severe maternal morbidities. GDM, hemorrhage, HTN, MMHC are not. Do the categories of outcomes and predisposing condition perhaps deserve separate analyses? There should at minimum be an acknowledgement of the mixed list. It is also notable that the list includes AFE (estimated 1/40,000 births) and hypertension (1/5 births). | We thank the reviewer for this recommendation. We added a few sentences to the beginning of the methods section and the literature review subsection that better describe why the analysis includes only nine morbidities, some severe and some not. |
| In the small proportion of outcomes that increase maternal costs in this analysis I don't see any reference to level of care -- use of intensive care rather than typical maternity postpartum stay, yet this would be an outcome of AFE, cardiac arrest, possibly hemorrhage, possibly sepsis. | We added a sentence explaining that we used the average cost estimates found in the literature. In addition, the cost for each outcome encompasses medical and nonmedical costs, where medical costs are primarily the more intensive use of hospital care during a delivery hospitalization. We also revised our example of “medical costs” to reflect this explanation. |
| There is a vulnerability in this model -- for example, hypertension is linked to cardiac arrest and renal disease, but the authors do not "amplify" the cost of hypertension by linking the outcomes attributed to these. An explanation for these omissions would be reasonable. The opportunity to build on this to create more complex models that would include amplifications should be mentioned. This manuscript offers an excellent precedent and warrants publication with its analysis as it stands. | We discuss the limitation that we do not account for comorbidities, which would “amplify” the cost of each outcome, under “Unaccounted-for interactions and secondary outcomes.” We added a sentence about the opportunity to address this limitation in future models. |
| While the authors mention in their second to last paragraph that it would have been "ideal" to examine costs by race and ethnicity, they do not disclose why they didn't. It is true, as they mention, that SMM is more common in Black birthing populations, as are their other morbidities of interest (hemorrhage, GDM, hypertension). The compounded impact of lost productivity, income, mental health toll etc. on a population already structurally marginalized may tell a very different story. Especially as there is data that MMHC are identified in non-Hispanic white people at a greater rate than in Hispanic or NHB populations due to underscreening of the latter -- that is, this analysis is vulnerable to structural racism due to the morbidities included: there are inherently more white patients in the MMHC group with massive cost associated with its sequelae, and more Black patients in the hypertension group with is nearly 3x lower cost. Perhaps in the interest of building the literature around cost of maternal morbidities this discrepancy can be acknowledged not unpacked, but the manuscript deserves a better explanation of why race wasn't investigated. | We thank the reviewer for this important comment. We added some of these considerations to the discussion section, indicating that there was not enough evidence in the literature of morbidity-outcome connections to conduct a subgroup analysis. We hope that future research – either by us or others – will measure costs of various maternal morbidity conditions by race and ethnicity. |
