# Supplementary material for: Societal cost of nine selected maternal morbidities in the United States
Source: PLoS One. 2022 Oct 26;17(10):e0275656. doi: 10.1371/journal.pone.0275656 (PMC9603953; doi:10.1371/journal.pone.0275656)
Supplement: Attachment — Submitted filename: Response to Reviewers.docx [file pone.0275656.s011.docx]

Sasigant So O’Neil, SM

Mathematica, P.O. Box 2393

Princeton, NJ 08543-2393

(617) 301-8975

[soneil@mathematica-mpr.com](mailto:soneil@mathematica-mpr.com)

Emily Chenette, PhD

Editor-in-Chief, PLOS ONE

1265 Battery Street, Suite 200

San Francisco, CA 94111, USA

September 20, 2022

Dear Dr. Chenette,

Thank you and the reviewers for providing additional comments on our manuscript on September 14th, 2022. We are now submitting the enclosed revised paper, “Societal Cost of Nine Selected Maternal Morbidities in the United States,” Manuscript # PONE-D-22-11649R1, for publication in *PLOS ONE*.

We revised our manuscript to address the clarifications raised by the reviewers, which are shown in the revised manuscript with track changes and described directly in the table below. We also include a clean version of the manuscript with this submission.

All authors have read and approved the revised manuscript. We hope that our resubmission is now suitable for inclusion in *PLOS ONE* and we look forward to hearing from you.

Sincerely,

Sasigant So O’Neil, SM

Director, Domestic Health Philanthropy Portfolio

Mathematica

| Reviewer #3 comment | Author Response |
| --- | --- |
| First, the study estimates costs over 5 years. Clearly the annual costs fall over this period, and it looks like most of the high costs consequences occur in this period. It would help if there were a clearer justification of the choice of 5 years, and a comment on the likely scale of underestimation of costs that result from this. | We discuss in the introduction that the five-year period of our model is most salient to policymakers. We added language to this effect under the Methods section explaining the modeling approach. |
| Second, i was a little unclear about the way in which health care inflation was used. There are two main reasons why costs in healthcare rise - general increases in pay and prices (which are not really relevant, since for decision making real rather than nominal costs are relevant), and increases in costs that are specific to the health sector, some of which are Baumol effects. The latter are relevant but are probably a small part of the increases. | To clarify, we added the following text to the Methods section explaining the modeling: “Following the U.S. Public Health Service’s recommendations for cost models, we discounted costs at an annual rate of 3% to reflect the lower economic value of future expenses and accounted for increases in prices of healthcare services and commodities across time. For medical costs, we used the medical care component of the Consumer Price Index to adjust prices to 2019 dollars. For non-medical costs, we used the Consumer Price Index for all items, less food and energy, to adjust prices to 2019 dollars We also included a medical inflation rate of 4.53%, based on the annual change in the medical care component of the Consumer Price Index from December 2018 to December 2019.” |
| Third, the co-morbidity point has been expanded, but i think we could still get a little more useful comment around this, particularly around the way in which costs for a single disease can be much higher in the context of co-morbidity. | We added text on page 16, lines 296-298, to address this: “In this example, the cost of delivery for a pregnant person with hypertension and gestational diabetes could end up substantially higher than the cost of delivery for a pregnant person with only one of these conditions.” |
| Fourth, it is commented that the outcomes in question have risen substantially - is it clear if this is a real increase or a change in reporting? | We have added text on page 18, lines 347-348 about this. The question of whether changes in the outcomes in question are real or due to reporting changes is beyond the scope of this study, but it is an important consideration. |
| Finally, and less seriously, it is a little odd to describe maternal deaths as one of the more serious outcomes. Some would argue it is the most serious! | We have revised the text on page 3, line 12 to say “Maternal mortality is the most serious consequence of maternal morbidity” |
